# Newly designed analogues from SARS-CoV inhibitors mimicking the druggable properties against SARS-CoV-2 and its novel variants†

**DOI:** 10.1039/d1ra04107j

**Published:** 2021-09-22

**Authors:** Nadim Ferdous, Mahjerin Nasrin Reza, Md. Shariful Islam, Md. Tabassum Hossain Emon, A. K. M. Mohiuddin, Mohammad Uzzal Hossain

**Affiliations:** Department of Biotechnology and Genetic Engineering, Faculty of Life Science, Mawlana Bhashani Science and Technology University Santosh Tangail-1902 Bangladesh; Department of Biology, University of Kentucky 101 T.H. Morgan Building Lexington KY 40506-022 USA; Bioinformatics Division, National Institute of Biotechnology Ganakbari, Ashulia, Savar Dhaka-1349 Bangladesh uzzalbge10044@gmail.com

## Abstract

The emerging variants of SARS coronavirus-2 (SARS-CoV-2) have been continuously spreading all over the world and have raised global health concerns. The B.1.1.7 (United Kingdom), P.1 (Brazil), B.1.351 (South Africa) and B.1.617 (India) variants, resulting from multiple mutations in the spike glycoprotein (SGp), are resistant to neutralizing antibodies and enable increased transmission. Hence, new drugs might be of great importance against the novel variants of SARS-CoV-2. The SGp and main protease (M^pro^) of SARS-CoV-2 are important targets for designing and developing antiviral compounds for new drug discovery. In this study, we selected seventeen phytochemicals and later performed molecular docking to determine the binding interactions of the compounds with the two receptors and calculated several drug-likeliness properties for each compound. Luteolin, myricetin and quercetin demonstrated higher affinity for both the proteins and interacted efficiently. To obtain compounds with better properties, we designed three analogues from these compounds and showed their greater druggable properties compared to the parent compounds. Furthermore, we found that the analogues bind to the residues of both proteins, including the recently identified novel variants of SARS-CoV-2. The binding study was further verified by molecular dynamics (MD) simulation and molecular mechanics/Poisson Boltzmann surface area (MM/PBSA) approaches by assessing the stability of the complexes. MD simulations revealed that Arg457 of SGp and Met49 of M^pro^ are the most important residues that interacted with the designed inhibitors. Our analysis may provide some breakthroughs to develop new therapeutics to treat the proliferation of SARS-CoV-2 *in vitro* and *in vivo*.

## Abstract 2

Three designed inhibitors with potential inhibition efficacy against the emerging variants of SARS coronavirus-2 (SARS-CoV-2).

## Graphical abstract

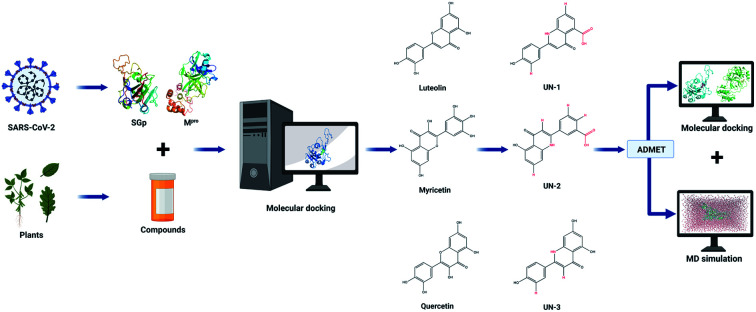

## Introduction

The first case of severe acute respiratory syndrome coronavirus 2 (SARS-CoV-2) was reported on December 30, 2019, in Wuhan city of Hubei province in China.^1–4^ The symptoms of COVID-19 caused by SARS-CoV-2 are usually fever, cough, sore throat, and breathlessness among others.^5^ The World Health Organization (WHO) announced a Public Health Emergency across the globe for this outbreak. The emergence of novel rapidly transmissible variants of SARS-CoV-2 threatens to prolong this pandemic, creating devastating health and economic consequences. This virus is now spread across 219 countries with a total of more than 137 million cases, at the date of writing. The situation of the South-East Asian countries is also frightening as most of the low-income countries of this region lack basic health care facilities, so they are failing to combat the pandemic. As of May 4th, Bangladesh has a death toll of 11 766 with 767 914 confirmed cases, being one of the most severely affected countries experiencing cases of the B.1.1.7 (ref. 6) and B.1.351 (ref. 7) variants in South-East Asia.

SARS-CoV-2 is a single-stranded positive-sense RNA virus and its genome size is ∼30 kb, which is the largest among all RNA viruses.^8^ SARS-CoV-2 entry into host cells is mediated by the spike (S) glycoprotein which comprises two functional subunits: one (the S1 subunit) is responsible for binding to the host cell receptor and the other (the S2 subunit) is responsible for fusion of the viral and cellular membranes.^9^ It is reported that SARS-CoV-2 utilizes human angiotensin-converting enzyme 2 (hACE2) as the receptor.^10^ Reports from fall 2020 revealed the presence of a D614G variant of SGp and it quickly became dominant.^11^ Recently some emerging variants with rapid transmission capability have been discovered in the UK, Brazil and South Africa. The UK variant (B.1.1.7) shares the N501Y mutation with the B.1.351 (South Africa) and the B.1.1.28 (Japan) variants responsible for causing improved affinity of the viral SGp with cellular receptors.^6,7^ The B.1.351 and P.1 (Brazil) variants (20H/501Y.V2) contain three SGp mutations, K417N, E484K, and N501Y.^12^ Another new lineage of SARS-CoV-2, B.1.617 with a combination of L452R and E484Q spike mutations has been reported in India.^13^ Recent studies have shown that the K417N and E484K mutations can enhance RBD–ACE2 binding, making them more transmissible.^14^ These two mutations also help the virus escape therapeutically relevant monoclonal antibodies (mAbs).^14^ Meanwhile, the UK variant has no obvious effect on mAbs but can also increase the RBD's binding affinity with ACE2.^14^ A recent study from California revealed that another mutation from the B.1.617 variant, L452R, can increase infectivity.^13^ All these mutations of SGp have been raising concerns about an increase in number of individuals re-infected by SARS-CoV-2 threatening the efficacy of current vaccines.

The main protease (M^pro^) of SARS-CoV-2, also called the 3-C like protease (3CL^pro^), consists of 306 amino acids and has 3 domains. Domain I and domain II contribute one residue to a catalytic dyad with Cys145 and His41, which is then connected by a long loop to domain III.^15^ It is one of the best characterized drug targets among coronaviruses because this enzyme is essential for processing the polyproteins that are translated from the viral RNA.^16^ Therefore, SGp and M^pro^ are attractive drug targets for designing novel inhibitors in order to prevent viral attachment and replication.

The process of drug discovery requires a multi-disciplinary effort to design effective and commercially feasible drugs against any pathogens. The objective of drug design is to find a chemical compound able to fit both geometrically and chemically to a specific cavity on a target protein.^17^ The chemical compounds can be found in nature or synthesized in laboratories. This compound becomes a drug available to patients after passing several animal tests and different phases of human clinical trials.^17^ But these methods are high cost and time consuming. In comparison, a modern approach including structure-based drug design with the help of computational and *in silico* methods can be carried out within a short period of time which is cost effective and has speeded up the drug discovery process. From the beginning of the COVID-19 pandemic, researchers and computational biologists have been trying to find the small molecule inhibitors that can restrict the proliferation of several proteins of SARS-CoV-2.^18–22^ Despite having a large number of natural compounds that can bind and interfere with the significant proteins of SARS-CoV-2, many of them possess toxic properties as well as being metabolically unstable; as a result, these compounds do not enter into experimental research over time in a wet laboratory. Also, most of the potent compounds were not able to bind with mutation-susceptible residues, so upon mutation occurring, the compounds might not inhibit the target proteins. Thus, designing non-toxic compounds from existing ones capable of inhibiting multiple variants of pathogens remains one of the most important challenges in computer aided drug design (CADD).

Addressing these challenges, we selected seventeen known phytochemicals to identify the potential candidates for designing novel inhibitors against SGp (wildtype and mutants) and M^pro^ of SARS-CoV-2 in this study. Luteolin, myricetin and quercetin showed better results in terms of binding with both SGp and M^pro^ and in drug-likeliness properties than the remaining compounds. Considering binding affinities, interactions with target proteins and the ADMET profile, we designed three novel inhibitors from these three compounds that outnumber all seventeen compounds in interfering with the function of both proteins including the SGp variants. Additionally, we have analyzed and validated the stability of protein–inhibitor complexes using MD simulation and the MM/PBSA approach. We also addressed the key residues of both proteins that are crucial for binding of compounds. As no known therapeutics are available for SARS-CoV-2 to date, the results of this study might be valuable references for further experimental research.

## Materials and methods

**Fig. 1:**
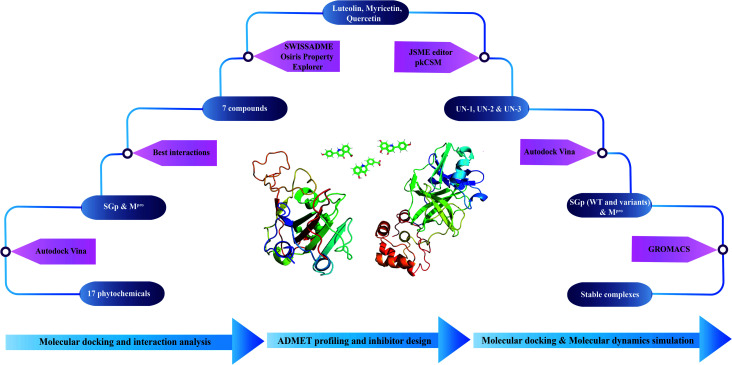
Schematic workflow of designing novel inhibitors against SGp and M^pro^ of SARS-CoV-2.

A step-wise protocol was followed to design the novel inhibitors against SGp and M^pro^ of SARS-CoV-2. The work flow is shown in Fig. 1.

### Selection and preparation of phytochemicals

Seventeen phytochemicals with antiviral activity were selected for this study. The phytochemicals are aloe-emodin, amentoflavone, apigenin, beta-sitosterol, betulonic acid, curcumin, hesperetin, hinokinin, indigo, isotheaflavin 3′-gallate, luteolin, myricetin, niclosamide, quercetin, savinin, scutellarein and theaflavin 3,3′-digallate. All these compounds were found to have inhibitory efficacy against several proteins of SARS-CoV that emerged in 2002.^23^ The Canonical SMILES of these seventeen phytochemicals were collected from the PubChem database and converted into protein data bank files (PDB) using the Online SMILES Translator and Structure File Generator (https://cactus.nci.nih.gov/translate/).

### Preparation of SGp and M^pro^

The structure of the spike receptor-binding domain (PDB ID: 6LZG) at 2.50 Å resolution and M^pro^ (PDB ID: 6LU7) bound to an inhibitor N3 (*N*-[(5-methylisoxazol-3-yl)carbonyl]alanyl-l-valyl-*N*∼1∼-((1*R*,2*Z*)-4-(benzyloxy)-4-oxo-1-{[(3*R*)-2-oxopyrrolidin-3-yl]methyl}but-2-enyl)-l-leucinamide) at 2.16 Å resolution of SARS-CoV-2 are available in the Research Collaboratory for Structural Bioinformatics (RCSB) Protein Data Bank (PDB). These structures were collected and the ligand molecules, hetero atoms and water molecules were removed from the structure using Discovery Studio 4.0 client (http://www.accelrys.com/products/discovery-studio/). Only chain B of 6LZG and chain A of 6LU7 were kept for further docking analysis. Later, energy minimizations were performed using SwissPdb Viewer^24^ and polar hydrogen atoms were added to all these proteins by employing AutoDock Tools.^25^

### Exploration of active site

The active sites of SGp and M^pro^ were explored with the Computed Atlas of Surface Topography of Protein (CASTp) (http://www.sts.bioengr.uic.edu/castp/) server which provides an online resource for locating, depicting and measuring concave surface regions on 3D structures of proteins.^26^

### Molecular docking of phytochemicals against wildtype (WT) SGp and M^pro^

Molecular docking simulations were executed with AutoDock tools to determine receptor–ligand interactions. The active site of SGp was enclosed with the parameters of a grid box with *X* = 34, *Y* = 34, *Z* = 88 (center grid box: *X* = −40.085, *Y* = 49.129, *Z* = 13.331; spacing = 0.347 Å) dimensions. In the case of the main protease (M^pro^), a grid box set to cover all the residues of the active site (center *X* = −11.796, *Y* = 21.447, *Z* = 72.229, dimensions *X* = 60, *Y* = 64, *Z* = 60 and spacing = 0.347 Å). (AutoDock Vina)^27^ was used to accomplish all the docking simulations with the predetermined parameters. Further, the receptor–ligand interaction was visualized using PyMOL 1.1 (ref. 28) and Discovery studio 4.0 client.

### Validation of molecular docking approach

As no respective inhibitors of SGp are available in the PDB database up to now, for validation of docking purposes, we first removed the N3 inhibitor from the M^pro^ (6LU7) and re-docked it in the active site of 6LU7 using AutoDock Vina to act as a positive control. The re-docked pose of N3 was then superimposed onto the reference co-crystallized N3 using PyMOL and the root mean square deviation (RMSD) was calculated. Afterwards, we analyzed the interactions of a positive control with M^pro^.

### Drug-likeliness prediction

Molecular properties based on Lipinski's rule of five and the bioavailability score of each phytochemical docked with both SGp and M^pro^ were calculated on SWISSADME.^29^ Drug scores were determined with the Osiris property explorer (https://www.organic-chemistry.org/prog/peo/).

### Novel inhibitor design

In order to improve the anti-COVID-19 activity of the phytochemicals, novel inhibitors were designed by generating their analogs. JSME structure editor, Osiris property explorer and SWISSADME were used to complete the steps. For the development of an accurate alignment of the side chains and stereochemistry of the designed molecules, all of them were implemented with the Yet Another Scientific Artificial Reality Application (YASARA) force field.^30^ YASARA conducted molecular corrections of the designed analogs which is vital for structural stability at the molecular level.^31–33^

### ADMET studies and molecular docking with SGp (WT and mutants) and M^pro^

The ADMET (absorption, distribution, metabolism, excretion and toxicity) properties of the designed analogs were determined with the pkCSM server^34^ and compared with the parent compounds. This is a freely accessible web server (http://biosig.unimelb.edu.au/pkcsm/) for analyzing *in silico* ADMET properties of compounds. The designed inhibitors were docked with SGp (WT and mutants) and M^pro^. Considering the rapid transmissibility, we selected the K417N, E484K, N501Y and L452R mutant SGp to test the inhibition efficacy of the designed compounds. The structure of the receptor binding domain of SARS-CoV-2 B.1.351 variant SGp in complex with COVOX-222 and EY6A Fabs (PDB ID: 7NXA) at 2.50 Å resolution is available in the PDB database. The structure was prepared by removing all the ligand molecules. As no known crystal structure of the B.1.617 variant of SGp was available in the database, we carried out the L452R and E484Q mutations in the WT structure (6LZG) using the mutagenesis tool of PyMOL. Further, site-specific docking was performed using Vina covering the four mutation sites. Each designed inhibitor was docked with the four SGp mutants and the results were analyzed.

### Molecular dynamics (MD) simulations and MM/PBSA calculation

Molecular dynamics (MD) simulations were performed to assess the structural stability of protein–inhibitor complexes at an atomistic level. The GROningen MAchine for Chemical Simulations (GROMACS) version 5.1.4 (ref. 35) was used to accomplish this task. The ‘pdb2gmx’ script was used to prepare the protein topologies while ligand topologies were generated from the PRODRG server.^36^ The energy-minimized conformations of the complexes were obtained with the GROMOS96 43a1 force field and then solvated with a single point charge (SPC) water model in a rectangular box where every structure was placed in the center at least 1.0 nm from the box edges. The ‘gmx genion’ script was used to neutralize the net charges in the systems. By employing the steepest descent-minimization algorithm, the energy minimization of all the complexes was undertaken with a maximum of 50 000 steps and <10.0 kJ mol^−1^ force. Afterwards, two steps were conducted to equilibrate the systems: an NVT (constant number of particles, volume, and temperature) ensemble and an NPT (constant number of particles, pressure, and temperature) ensemble. Both the NVT and the NPT series were conducted at 300 K temperature and 1 atm for a duration of 100 picoseconds (ps). V-rescale was selected as the thermostat and Parrinello–Rahman was selected as the barostat of the performed simulation. Finally, the production simulation was performed at 300 K for a duration of 120 nanoseconds (ns) in the supercomputing system of the National Institute of Biotechnology, Savar, Bangladesh with a time step of 2 fs, and the structural coordinates were saved after every 10 ps. Thereafter, root mean square deviation (RMSD), root mean square fluctuation (RMSF), radius of gyration (*R*_g_), number of hydrogen bonds and solvent accessible surface area (SASA) were analyzed to evaluate the stability of the complexes. The graphs were plotted using GRACE software. Further, the MM/PBSA binding free energies were calculated using the ‘g_mmpbsa’^37^ package of GROMACS followed by a final MD run. The binding energies in this method were calculated with the following equation:Δ*G*_binding_ = *G*_complex_ − (*G*_protein_ + *G*_ligand_)Here, Δ*G*_binding_ = the total binding energy of the complex, *G*_protein_ = the binding energy of free protein, and *G*_ligand_ = the binding energy of unbounded ligand.

## Results

### Predicted active site

The active site regions of the SGp and M^pro^ of SARS-CoV-2 were identified with the CASTp server. The preeminent active site of SGp was found in areas with 74.091 and a volume of 32.521 amino acids whereas the best active site of M^pro^ was found in areas with 351.125 and a volume of 319.370 amino acids.

### Molecular docking analysis of phytochemicals with SGp and M^pro^

**Fig. 2:**
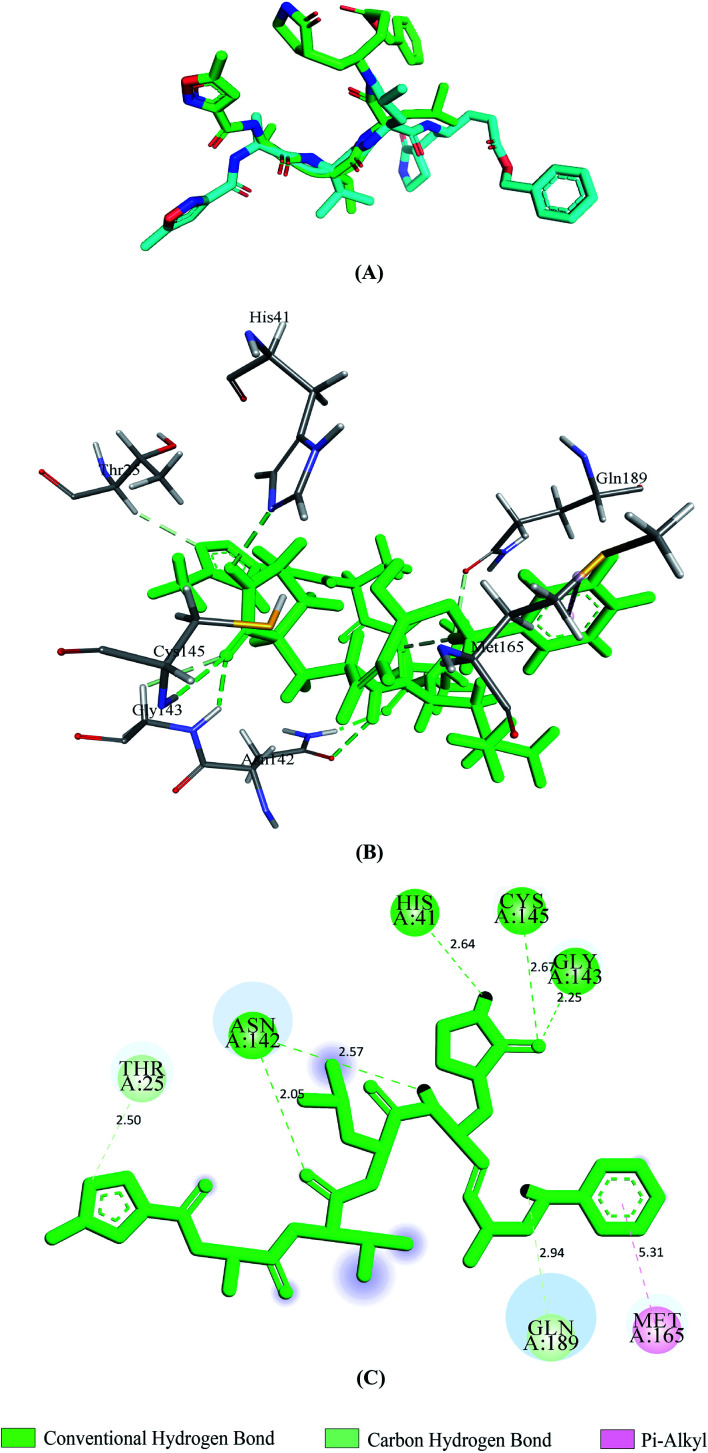
Validation of docking approach by re-docking N3 with 6LU7. Superimposition of re-docked pose of N3 (light green) and the co-crystallized N3 (cyan) from 6LU7 (A), 3D and 2D images depicting interactions (B and C) between N3 and M^pro^.

Vina predicted nine possible binding positions as output for each compound. Out of nine possible ligand binding positions, the best one was chosen for each compound based on the lowest docking energy. The docking energy score of all seventeen phytochemicals with both SGp and M^pro^ are shown in Table S1.† The amino acid interactions of both proteins with these phytochemicals were also identified. Twelve phytochemicals out of seventeen showed favorable interaction with the active site pocket of both SGp and M^pro^ and seven were bonded to at least five active amino acid residues of both proteins (Table 1). These seven phytochemicals were considered for further analysis, excluding the rest. The selected phytochemicals presented lower docking energy ranging from −6.1 to −8.9 kcal mol^−1^. The N3 showed a docking energy of −6.9 kcal mol^−1^ with M^pro^. The re-docked N3 was then superimposed onto the native co-crystallized N3 using PyMOL and a low RMSD of 0.226 Å was observed. From the interaction analysis shown in Fig. 2, we found that the control N3 had similar interaction with M^pro^ to that we noticed in the interactions of the co-crystallized N3–M^pro^ complex. Thr25, His41, Asn142, Gly143, Cys145, Met165 and Gln189 are the interacting amino acids in the active site pocket and about five hydrogen bonds were formed. All the interacted residues of M^pro^ with the control were active site residues and almost all of our selected compounds interacted with these similar residues.

**Table 1:** Molecular docking results of the selected seven phytochemicals with SGp and M^pro^

Phytochemicals	Binding energy with SGp (kcal mol^−1^)	Interacted residues of SGp	Binding energy with M^pro^ (kcal mol^−1^)	Interacted residues of M^pro^
Aloe-emodin	−6.1	Arg454, Phe456, Arg457, Lys458, Asp467, Glu471	−7.5	His41, Leu141, Asn142, Cys145, Glu166, Arg188
Isotheaflavin 3′-gallate	−7.2	Arg454, Arg457, Lys458, Asp467, Ser469	−7.2	Thr26, His41, Ser46, Ser144, Leu141, Asn142, Cys145, His163, Glu166
Luteolin	−7.0	Arg454, Arg457, Lys458, Asp467, Ser469, Glu471	−7.4	His41, Asn142, Cys145, Arg188, Thr190, Gln192
Myricetin	−6.3	Arg454, Arg457, Lys458, Asp467, Glu471	−7.4	Leu141, Gly143, Ser144, Cys145, His163, Met165
Niclosamide	−6.2	Arg454, Arg457, Lys458, Asp467, Ser469, Glu471	−7.0	Thr26, Leu141, Gly143, Ser144, Cys145, Glu166
Quercetin	−6.3	Arg454, Arg457, Lys458, Asp467, Ser469, Glu471	−7.3	Leu141, Gly143, Ser144, Cys145, His163, Met165, Arg188
Theaflavin 3,3′-digallate	−6.5	Arg454, Phe456, Arg457, Lys458, Asp467, Ser469, Glu471	−8.9	His41, Ser46, Leu141, Gly143, Ser144, Cys145, Met165, Glu166, Gln189, Thr190

### Predicted drug-likeliness properties

The SwissADME web server calculated properties based on Lipinski's rule of five and the bioavailability score, while the drug score was determined from the Osiris Property Explorer, as shown in Table 2. Based on higher bioavailability and drug score (bioavailability score >0.50 and drug score >0.30), three phytochemicals, luteolin, myricetin and quercetin, were chosen. Further, these three phytochemicals were used as lead compounds to generate three novel analogue inhibitors.

**Table 2:** Bioavailability score and drug score of the selected phytochemicals

Phytochemicals	Lipinski filter	Bioavailability score	Drug score
Aloe-emodin	Yes; 0 violation	0.55	0.21
Isotheaflavin 3′-gallate	No; 3 violations: MW > 500, N or O > 10, NH or OH > 5	0.17	0.39
Luteolin	Yes; 0 violation	0.55	0.84
Myricetin	Yes; 1 violation: NH or OH > 5	0.55	0.46
Niclosamide	Yes; 0 violation	0.55	0.14
Quercetin	Yes; 0 violation	0.55	0.30
Theaflavin 3,3′-digallate	No; 3 violations: MW > 500, N or O > 10, NH or OH > 5	0.17	0.31

### Designed novel inhibitors and ADMET profiling

**Fig. 3:**
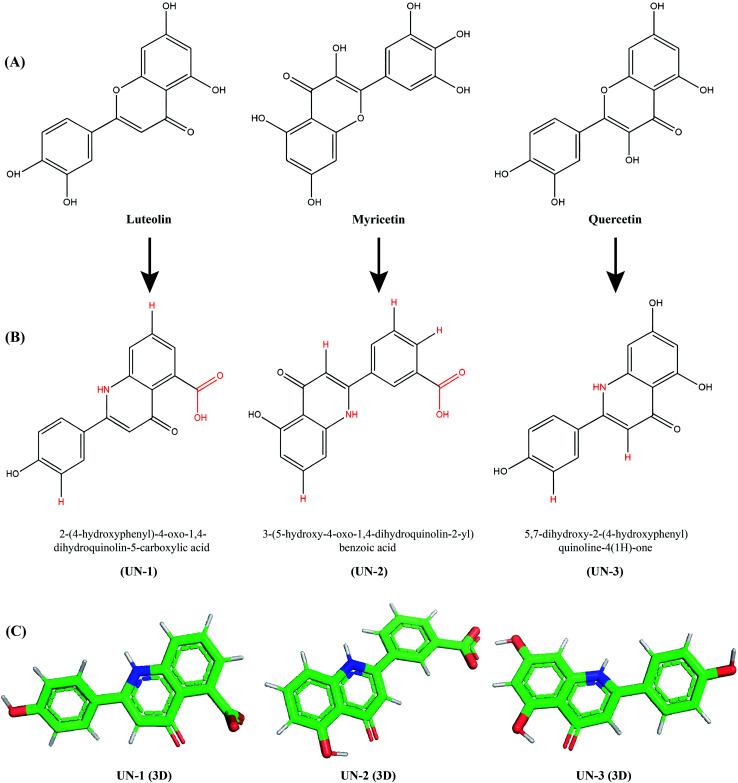
Parent compounds and newly designed analogues: (A) three parent compounds, (B) three designed analogues drawn in ChemDraw Ultra 12.0 (changed groups are shown in red), (C) energy-minimized three-dimensional structures of the designed analogues visualized in PyMOL.

To improve the binding affinity and ADMET properties of the three selected compounds, we designed three analogues named UN-1 (2-(4-hydroxyphenyl)-4-oxo-1,4-dihydroquinolin-5-carboxylic acid), UN-2 (3-(5-hydroxy-4-oxo-1,4-dihydroquinolin-2-yl)benzoic acid) and UN-3 (5,7-dihydroxy-2-(4-hydroxyphenyl)quinoline-4(1*H*)-one). Several groups have been replaced with NH, COOH and H groups to generate these analogues, as shown in Fig. 3. The ADMET properties of the designed inhibitors were analyzed for their drug likeliness, drug score, toxicity, bioavailability *etc.*, as shown in Table 3. Among the ADMET properties, the percentage of human intestinal absorption rate has increased for all three designed inhibitors, that is about 94% for both UN-1 and UN-2, about 81% for UN-3. All the compounds show Caco-2 permeability between 0.24 and 0.998 log Papp in 10^−6^ cm s^−1^. No compound seems to cross the blood–brain barrier, as revealed by their negative scores ranging from −0.894 to −1.136. Also, no compound appears to inhibit hERG I, II receptors. Myricetin and quercetin were predicted to be mutagenic and the latter was also predicted to show tumorigenicity whereas the designed analogues did not show any of these effects. A PAINS alert confirmed the presence of catechol moieties in the parent compounds whereas no designed inhibitors had any such moieties in their structure. The synthetic accessibility values have significantly decreased in all the designed inhibitors. The bioavailability score has increased for UN-1 and UN-2. In contrast, drug scores have increased for UN-2 and UN-3. All these results indicate that UN-1, UN-2 and UN-3 are more likely to be used as inhibitors than their parent compounds in terms of pharmacological properties.

**Table 3:** Comparison of ADMET properties, medicinal chemistry profile and drug likeliness of the three designed analogues with their parent compounds

Properties	Luteolin	UN-1	Myricetin	UN-2	Quercetin	UN-3
**Absorption**						
Intestinal absorption (human)	81.13	94.757	65.93	94.506	77.207	81.577
Caco2 permeability	0.096	0.687	0.095	0.998	−0.229	0.24
*P*-Glycoprotein substrate	Yes	Yes	Yes	Yes	Yes	Yes
*P*-Glycoprotein I inhibitor	No	No	No	No	No	No
*P*-Glycoprotein II inhibitor	No	No	No	No	No	No

**Distribution**						
Fraction unbound (human)	0.168	0.186	0.238	0.121	0.206	0.123
BBB permeability	−0.907	−1.032	−1.493	−0.894	−1.098	−1.136
CNS permeability	−2.251	−2.231	−3.709	−2.34	−3.065	−2.327

**Metabolism**						
Inhibitory substrate to	CYP1A2, CYP2C9	CYP1A2	CYP1A2	CYP1A2	CYP1A2	CYP1A2, CYP2C9

**Excretion**						
Total clearance	0.495	0.592	0.422	0.671	0.407	0.545
Renal OCT2 substrate	No	No	No	No	No	No

**Toxicity**						
AMES toxicity	No	No	No	No	No	No
hERG I inhibition	No	No	No	No	No	No
hERG II inhibition	No	No	No	No	No	No
Mutagenicity	No	No	Yes	No	Yes	No
Tumorigenicity	No	No	No	No	Yes	No

**Medicinal chemistry profile**						
PAINS	1 alert: catechol_A	0 alert	1 alert: catechol_A	0 alert	1 alert: catechol_A	0 alert
Brenk	1 alert: catechol	0 alert	1 alert: catechol	0 alert	1 alert: catechol	0 alert
Lead-likeliness	Yes	Yes	Yes	Yes	Yes	Yes
Synthetic accessibility	3.02	2.17	3.27	2.41	3.23	2.25

**Drug likeliness**						
Bioavailability score	0.55	0.56	0.55	0.56	0.55	0.55
Drug score	0.84	0.80	0.46	0.51	0.30	0.85

**Table 4:** Energy minimization score of newly designed inhibitors, UN-1, UN-2 and UN-3

Inhibitors	Start energy (kJ mol^−1^)	End energy (kJ mol^−1^)
UN-1	−294.0	−331.8
UN-2	−677.9	−735.4
UN-3	−711.7	−827.7

### Molecular docking analysis of designed inhibitors of SGp (WT and mutants) and M^pro^

**Fig. 4:**
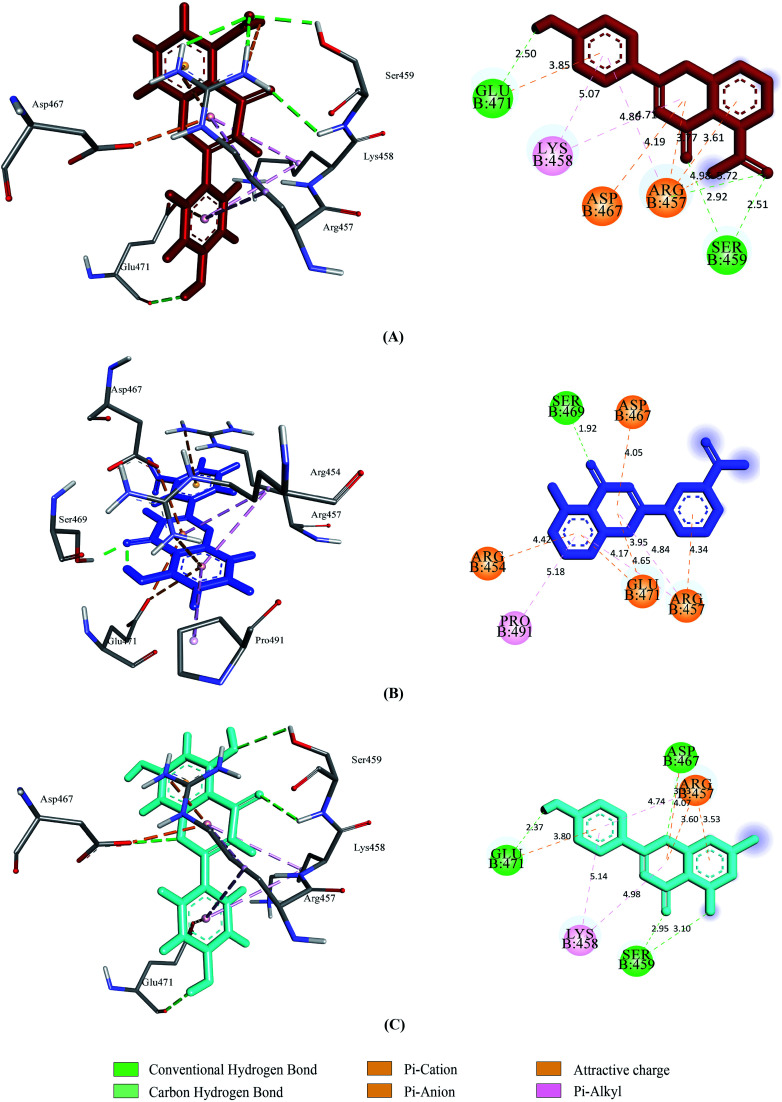
2D and 3D representation of molecular docking analysis between the SARS-CoV-2 SGp with (A) UN-1, (B) UN-2, (C) UN-3.

**Fig. 5:**
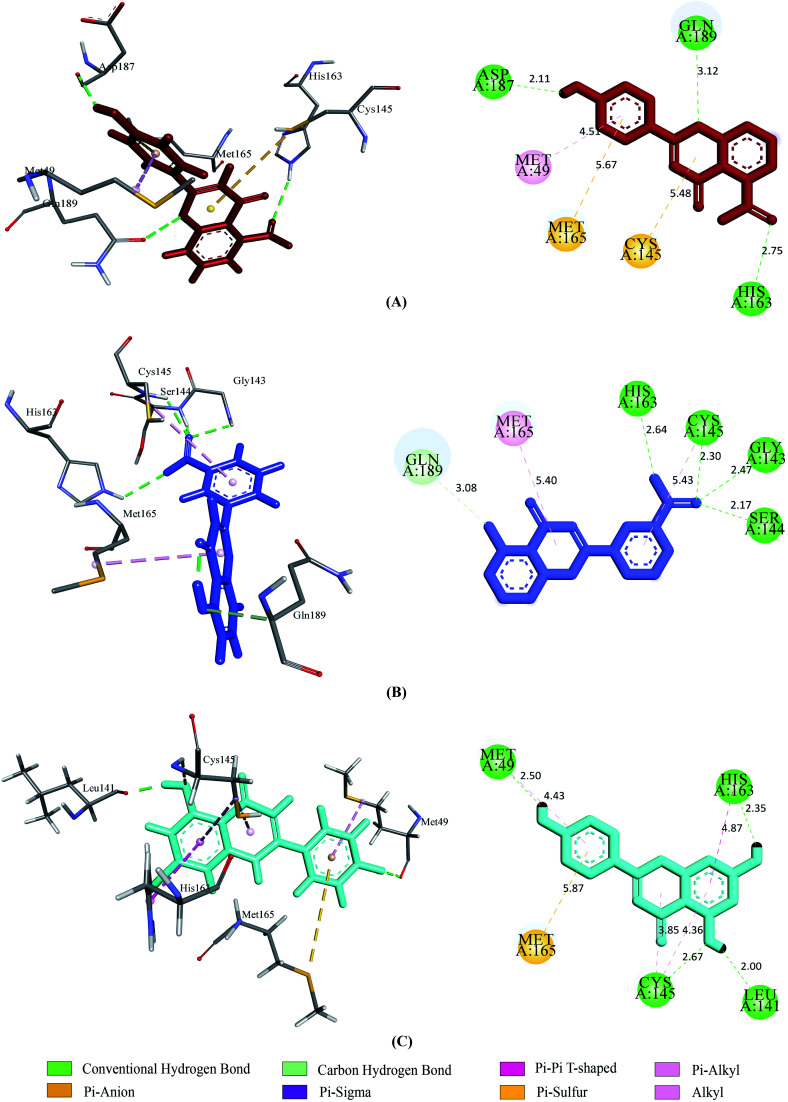
2D and 3D representation of molecular docking analysis between the SARS-CoV-2 M^pro^ with (A) UN-1, (B) UN-2, (C) UN-3.

**Fig. 6:**
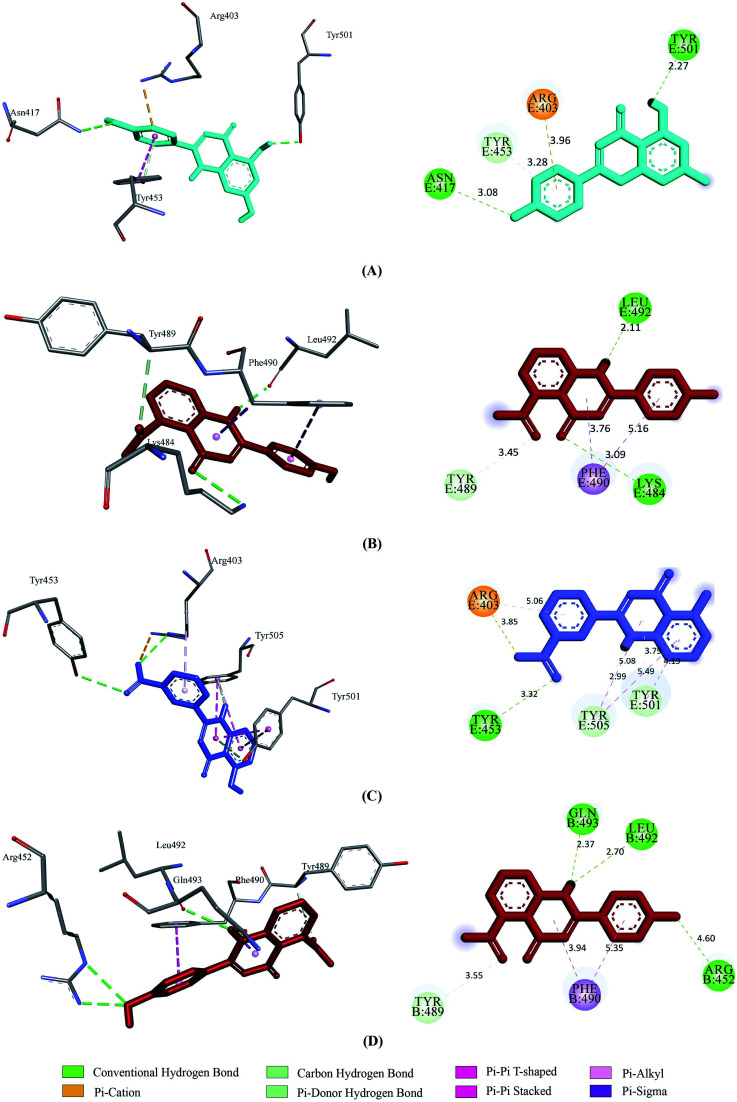
2D and 3D representation of molecular docking analysis between the SARS-CoV-2 SGp mutants and designed inhibitors, (A) SGp(K417N)-UN-3, (B) SGp(E484K)-UN-1, (C) SGp(N501Y)-UN-2, (D) SGp(L452R)-UN-1.

Prior to the molecular docking simulation of the three designed inhibitors, the YASARA energy minimization server was employed to minimize the energy of each compound. An energy comparison between all three designed ligands from the START energy to END energy confirms the energy minimization of the structural features (Table 4). The results of binding from Vina show that the three designed inhibitors bind to both SGp (WT) and M^pro^ with higher affinity than the parent compounds. UN-1, UN-2 and UN-3 exhibited docking scores of −7.0 kcal mol^−1^, −5.9 kcal mol^−1^ and −6.5 kcal mol^−1^ towards SGp. It was observed that Arg457, Ser459 and Asp467 were common participant residues in all SGp–inhibitor interactions (Fig. 4). The highest number of hydrogen bonding interactions was observed in the quercetin–SGp complex with Ser459, Asp467 and Glu471 residues. Ser459 also yielded hydrogen bonding with the other two complexes. The three designed inhibitors showed −7.5 kcal mol^−1^, −6.9 kcal mol^−1^ and −7.9 kcal mol^−1^ binding energy towards M^pro^. The common interaction residues include His163, Cys145 and Met165 (Fig. 5). Four hydrogen bonding interactions were observed in the cases of UN-2 and UN-3 whereas three were formed in the case of UN-1 with the active site residues of M^pro^. From a docking analysis of the designed inhibitors against the four SGp mutants, it was found that UN-1 showed higher binding affinities of −6.3 kcal mol^−1^ and −6.1 kcal mol^−1^ against the E484K and L452R mutants than against the other two designed inhibitors. UN-2 and UN-3 had higher binding affinities of −6.8 kcal mol^−1^ and −5.9 kcal mol^−1^ against the N501Y and K417N mutants. The N417, K484 and R452 mutant residues formed hydrogen bonding with the inhibitors where the highest number of hydrogen bonds was formed in the binding of UN-1 with the L452R variant (Fig. 6). The physicochemical and ligand binding properties of the designed compounds with SGp (WT) and M^pro^ are shown in Table S2.† The docking results of the designed compounds with SGp variants are shown in Table S3.†

### Molecular dynamics simulation

The molecular dynamics simulation of the ten protein–inhibitor complexes was performed for a period of 120 ns to analyze the physical movement of atoms at the atomistic level. The dynamic behavior of a protein can give rise to conformational changes which might affect its actual biological functioning.^38^ So, in order to examine the dynamic behavior and stability of the complexes throughout the 120 ns simulation period, all ten complexes were analyzed by calculating the RMSD, RMSF, *R*_g_, SASA and hydrogen bonds, as shown in Table 5.

**Table 5:** Average values of RMSD, RMSF, *R*_g_, SASA and number of hydrogen bonds of the ten protein–inhibitor complexes

Complex	RMSD (nm)	RMSF (nm)	*R*_g_ (nm)	SASA (nm^2^)	Number of hydrogen bonds
SGp-UN-1	∼0.34	∼0.41	1.77	∼96	∼3
SGp-UN-2	∼0.32	∼0.27	1.81	∼97	∼1
SGp-UN-3	∼0.28	∼0.28	1.78	∼98	∼3
SGp(K417N)-UN-3	∼0.23	∼0.26	∼1.78	∼95	∼1
SGp(E484K)-UN-1	∼0.21	∼0.16	∼1.78	∼98	∼2
SGp(N501Y)-UN-2	∼0.18	∼0.23	∼1.79	∼98	∼2
SGp(L452R)-UN-1	∼0.14	∼0.23	∼1.79	∼101	∼0.5
M^pro^-UN-1	∼0.33	∼0.39	2.11	∼133	∼2
M^pro^-UN-2	∼0.26	∼0.63	2.13	∼132	∼2
M^pro^-UN-3	∼0.29	∼0.40	2.14	∼138	∼3

### Root mean square deviation (RMSD) and root mean square fluctuation (RMSF) analysis

**Fig. 7:**
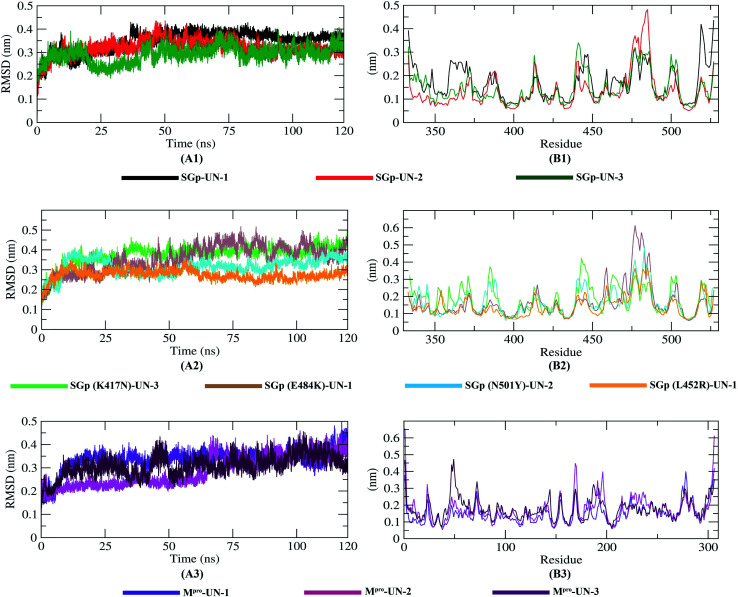
The RMSD and RMSF of Cα atoms of protein-inhibitor complexes. RMSD and RMSF graph of SGp(WT)–inhibitor complexes (A1–B1), SGp(mutant)–inhibitor complexes (A2–B2) and M^pro^–inhibitor (A3–B3) complexes from the molecular simulation of 120 ns at 300 K.

The dynamic movements of atoms and conformational variations of Cα backbone atoms of the ten complexes were calculated with RMSD to detect their stability. It can be observed from Fig. 7 that the SGp(L452R)-UN-1 complex exhibits a lower RMSD than the other nine complexes. The RMSD of SGp(WT)–inhibitor complexes (Fig. 7A1) increases from 50 to 75 ns; however, these values significantly decrease after 80 ns. While assessing the RMSD of SGp(mutant)–inhibitor complexes, a steady increase in RMSD is observed in the SGp(E484K)-UN-1 complex after 50 ns. Nonetheless, deviations for inhibitors complexed to other mutants were less than 0.5 nm (Fig. 7A2). Unlike the SGp–inhibitor complexes, M^pro^-UN-3 showed a consistent fluctuation over the 35–90 ns period, indicating that UN-3 might change the protein conformation. The remaining two inhibitors bound to M^pro^ exhibited a similar trajectory in the 75–100 ns period and tended to stabilize after 100 ns (Fig. 7A3).

To better understand the regions of proteins that are fluctuating during the simulation, the flexibility of each residue was calculated in terms of RMSF to get a better insight into the extent to which the binding of the designed inhibitors affects the flexibility of the proteins. It can be understood from Fig. 7B3 that binding with UN-3 makes M^pro^ most flexible in all areas in contrast to other complexes. UN-2 is found to induce local flexibility at Met49, Glu47, Asn72, Asp187, Arg188, Gln192 and Gly302. The SGp(E484K) structure is found to have the lowest RMSF, which indicates that the protein is not very flexible upon binding with UN-1. Besides, the RMSF values of both the WT and mutant SGp complexes are mostly similar in all areas. Overall, residues such as Ser371, Pro384, Thr385, Lys386, Lys444, Val445, Gly446, Pro463, Phe464, Glu465, Ser477, Asn501 and Val503 are found to be flexible for all the inhibitor-bound SGp complexes.

### Radius of gyration (*R*_g_) and solvent accessible surface area (SASA)

**Fig. 8:**
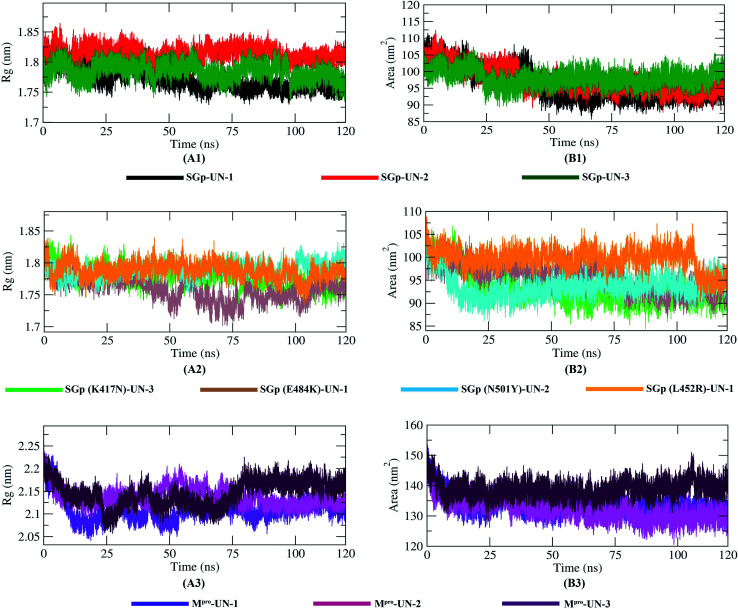
Radius of gyration (*R*_g_) plot reflecting the compactness of protein–inhibitor complexes (A1–A3) and SASA plot showing the variation in the solvent accessibility of the complexes (B1–B3) during the 120 ns MD simulations.

The compactness of the protein–inhibitor complexes is represented by the radius of gyration (*R*_g_). The greater compactness of a system is indicated by the lower degree of fluctuation throughout the simulation period. The *R*_g_ of WT SGp-UN-1 (Fig. 8A1) was found to be the lowest, indicating greater rigidity in contrast to the other complexes. Besides, a higher *R*_g_ of SGp-UN-2 was observed compared to the other two SGp–inhibitor complexes. Phenomena such as protein folding or unique conformational changes result in a greater change in *R*_g_. Inhibitor-bound K417N, N501Y and L452R mutant SGp complexes showed similar *R*_g_ throughout the simulation, while inconsistency was observed in the case of the SGp(E484K)-UN-1 complex. The three M^pro^–inhibitor complexes showed increased *R*_g_ from 10 to 75 ns, and the final parts of the trend were consistent. Although the *R*_g_ value of M^pro^-UN-3 was much higher after 75 ns, indicating the looseness of its packing compared to all the other complexes.

Interaction between protein–inhibitor complexes and solvents can be measured with the solvent accessible surface area (SASA) over the simulation time. So, the SASAs of all ten protein–inhibitor complexes were calculated to predict the extent of the conformational changes occurring during the interaction. Plots of SASA value *vs.* time for all the protein–inhibitor complexes are shown in Fig. 8. Interestingly, SGp(WT)-UN-3, SGp(L452R)-UN-1 and M^pro^-UN-3 featured an expansion of the surface area, showing relatively higher SASA values than the other complexes. The other inhibitor complexes did not increase in volume during the simulation process but remained in a stable state.

### Hydrogen bond analysis

**Fig. 9:**
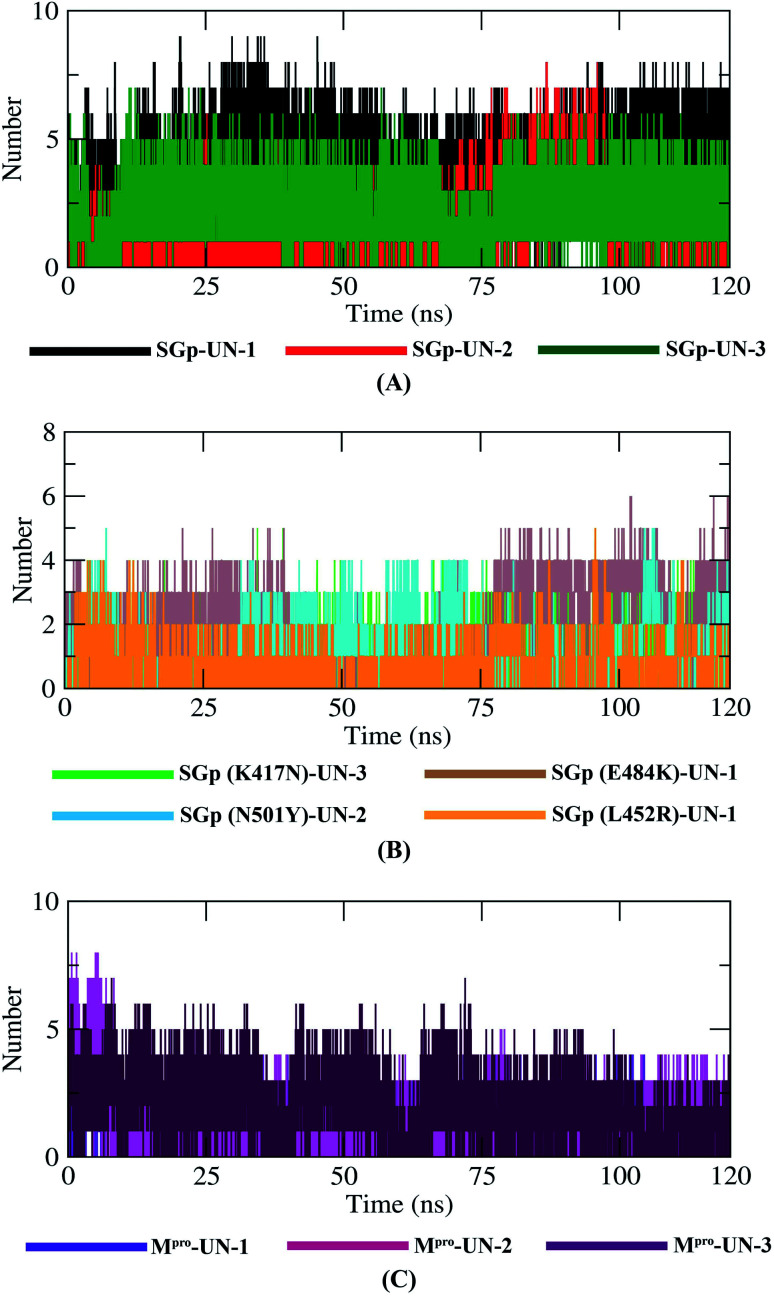
Plot representing the dynamics observed in the hydrogen bonding patterns for the SGp(WT)–inhibitor (A), SGp(mutant)–inhibitor (B) and M^pro^–inhibitor (C) complexes.

Hydrogen bonding between a protein–inhibitor complex is essential to stabilize the protein structures. Fig. 9 displays the total number of hydrogen bonds present in all ten complexes calculated after the 120 ns production run. It was observed that the highest number of conformations of SGp(WT) complexes formed up to five hydrogen bonds, mutant SGp complexes formed up to two hydrogen bonds and M^pro^ complexes formed up to four hydrogen bonds throughout the simulation. UN-1 and UN-2 formed the highest number of hydrogen bonds with SGp(WT) and M^pro^, respectively, during the simulation period. Interestingly, UN-1 and UN-2 also formed the highest number of hydrogen bonds with E484K and N501Y mutant SGp complexes.

### Post dynamics binding free energy results

**Fig. 10:**
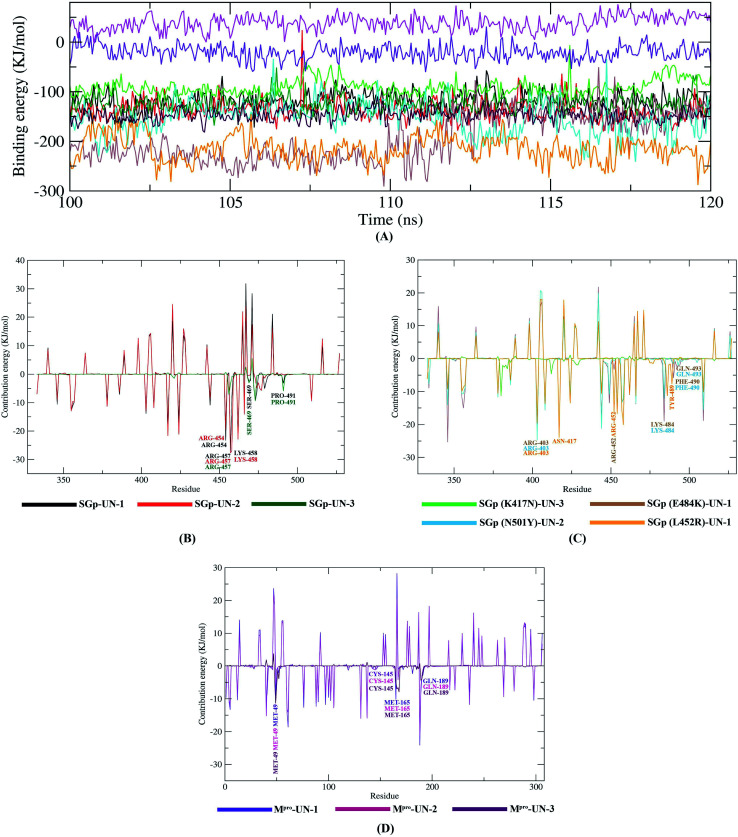
Graphical representation of the binding free energy of protein–inhibitor complexes (A) and per residue contribution plot for SGp–inhibitors (B), SGp(mutant)–inhibitors (C), M^pro^–inhibitors (D) complexes.

The binding free energy (MM/PBSA) of the last 20 ns with an interval of 50 ps was calculated from MD trajectories using the MM/PBSA method. Apart from the M^pro^-UN-2 complex, all the complexes showed negative total binding energies, indicating strong binding of the designed compounds with the target proteins (Table 6). In particular, SGp(L452R)-UN-1 complex depicted the lowest binding free energy and a higher binding affinity (−214.155 ± 26.323 kJ mol^−1^), suggesting a more stable conformation of UN-1. The remaining complexes also have favorable binding energies and, thus, these designed inhibitors could be used as potential lead compounds. A comparison of the binding free energies of all ten complexes was made by plotting the binding energy *versus* time graphs shown in Fig. 10.

**Table 6:** Binding free energy calculations (MM/PBSA) for ten protein–inhibitor complexes

Complex	van der Waals energy (kJ mol^−1^)	Electrostatic energy (kJ mol^−1^)	Polar solvation energy (kJ mol^−1^)	SASA energy (kJ mol^−1^)	Binding energy (kJ mol^−1^)
SGp-UN-1	−153.355 ± 14.684	−298.188 ± 32.518	345.938 ± 36.742	−13.150 ± 0.636	−118.756 ± 18.878
SGp-UN-2	−96.001 ± 8.894	−104.597 ± 35.037	71.040 ± 42.894	−7.920 ± 0.863	−137.478 ± 20.273
SGp-UN-3	−184.212 ± 11.486	−45.100 ± 9.213	120.643 ± 16.284	−14.204 ± 0.853	−122.874 ± 16.319
SGp(K417N)-UN-3	−111.776 ± 11.526	−32.936 ± 8.607	63.459 ± 14.781	−9.823 ± 0.737	−91.076 ± 15.572
SGp(E484K)-UN-1	−93.881 ± 25.697	−349.662 ± 86.527	258.331 ± 110.155	−10.181 ± 2.068	−195.394 ± 46.276
SGp(N501Y)-UN-2	−75.876 ± 16.915	−186.460 ± 65.676	117.115 ± 74.476	−8.016 ± 1.498	−153.236 ± 31.770
SGp(L452R)-UN-1	−97.354 ± 15.815	−181.414 ± 60.448	73.267 ± 65.259	−8.654 ± 1.224	−214.155 +− 26.323
M^pro^-UN-1	−197.339 ± 11.919	126.365 ± 31.436	64.367 ± 27.287	−14.173 ± 0.782	−20.780 ± 15.306
M^pro^-UN-2	−139.783 ± 11.157	139.668 ± 23.276	50.376 ± 26.112	−11.851 ± 0.864	38.410 ± 14.909
M^pro^-UN-3	−180.918 ± 12.496	−32.152 ± 14.250	80.529 ± 18.979	−14.278 ± 0.898	−146.820 ± 12.549

Further, we determined the contribution of each residue of SGp and M^pro^ in terms of binding free energy to the interaction with the designed inhibitors which was performed by decomposing the total binding free energy of the system into per residue contribution energy (Fig. 10).

Out of the residues showing interaction in molecular docking analysis, the Arg457 residue of SGp exhibited the highest contribution energies of −26.9792 kJ mol^−1^, −27.6262 kJ mol^−1^ and −3.6108 kJ mol^−1^, respectively, with UN-1, UN-2 and UN-3. Several residues of M^pro^ (Met49, CYS145, Met165 and Gln189) also showed favorable contribution energies in interacting with the active site pocket. The Met49 residue showed −9.27 kJ mol^−1^ contribution energy in complexes with UN-1, UN-2 and UN-3: the contribution energies were −5.05 kJ mol^−1^ and −11.4 kJ mol^−1^, respectively. Many residues of the four mutants showed favorable contribution energies towards the inhibitors. On comparing the complexes, we found that the mutant K484 and R452 residues involved in the interaction showed −16.8163 kJ mol^−1^ and −19.0742 kJ mol^−1^ contribution energies in complex with UN-1. This suggests that the binding of UN-1 to these mutants could interfere with the binding of SGp, as the residues would not be available to interact with ACE2. The contribution energies of other key residues of SGp (both WT and mutants) and M^pro^ are shown in Tables S4 and S5.†

## Discussion

Computer aided drug design (CADD) approaches have been utilized very widely in order to increase the efficiency of the course of drug discovery and development. This method requires three-dimensional (3D) structures of the target proteins, where the binding mode, affinity and confirmation of a ligand binding can be detected. Several CADD approaches are being evaluated as promising techniques in modern drug discovery. The key parts in CADD are molecular docking and molecular dynamics simulation that can be used to perform virtual screening of compounds to analyze how the ligands bind and inhibit a target and confirm their stability towards the target proteins.^39^ Also, the increasing development of robust computational tools allows scientists to assess the pharmacological properties of compounds before entering into experimental research. So, intensive CADD methods were used in this study to design novel inhibitors against COVID-19.

In this study, we emphasized both the binding affinity and ADMET properties of compounds to design potent inhibitors of SGp and M^pro^. As some newly emerged variants have recently been discovered that are mutated in various regions, we tried to design inhibitors capable of binding with both the key active site residues and the mutant residues of two proteins, so that these designed inhibitors can inhibit several strains of SARS-CoV-2. For this, seventeen phytochemicals with antiviral effects were selected and docked against the two proteins. Among them, twelve candidates were selected according to their high binding affinity towards the two proteins. It was found that isotheaflavin 3′-gallate exhibits the highest binding affinity of −7.2 kcal mol^−1^ while binding with SGp (Table S1†). Conversely, theaflavin 3,3′-digallate exhibits the highest affinity of −8.9 kcal mol^−1^ and interacted with the highest number of amino acid residues in the active site of M^pro^. As we addressed earlier, despite the high binding affinities of these compounds, we further filtered them based on Lipinski's rule of five and their bioavailability score to retain the biologically active compounds for further analysis (Table 2). Luteolin, myricetin and quercetin passed this stage and were considered as lead compounds to design novel inhibitors against the two significant proteins of SARS-CoV-2. To our knowledge, this is the first study to design novel inhibitors from these three flavonoids *via in silico* approaches against both SGp and M^pro^.

Among the selected three compounds, luteolin and quercetin, proved their capacity to block the entry of SARS-CoV in a dose-dependent manner (EC_50_ value of 10.6 μM) and quercetin was shown to prevent HIV-luc/SARS pseudotyped virus entry in host cells (EC_50_ of 83.4 μM). Myricetin showed an inhibitory effect against SARS-CoV NSP13 ATPase with IC_50_ values of 2.71.^40^ A study showed that luteolin is toxic to human lung embryonic fibroblasts (TIG-1) and human umbilical vein endothelial (HUVE) cells and quercetin was more toxic to HUVE cells than several flavonoids.^41^ Our computational analysis using up-to-date online-based tools also revealed several toxic properties, such as mutagenicity and tumorigenicity, as well as low intestinal absorption, an inhibitory effect to several isoforms of cytochrome P450 enzyme, and their possession of catechol groups (Table 3). Bearing in mind an improvement in these properties, we designed three analogues named UN-1, UN-2 and UN-3 and tested their binding affinity towards the two proteins and profiled their ADMET properties.

We found a significant increase in the intestinal absorption rate for all three analogues and they do not cross the blood–brain barrier. UN-1 does not inhibit the CYP2C9 isoform of cytochrome P450 enzyme unlike its parent compound, which is an important detoxification enzyme of the human body. No designed inhibitor shows mutagenic or tumorigenic properties like myricetin or quercetin. There is a very low chance of acquiring long QT syndrome from these designed inhibitors, but none appears to inhibit hERG I, II receptors (Table 3). A significant improvement was also observed in the medicinal chemistry profile of these designed inhibitors. Pan-assay interference compounds (PAINS) are chemical compounds that tend to give false positive results in high-throughput screens and react nonspecifically with several biological targets. Luteolin, myricetin and quercetin had a catechol group in their structures but it was not present in the designed inhibitors, as revealed by the PAINS filter. All the newly designed inhibitors have a low score of synthetic accessibility which denotes that these compounds can be synthesized much more easily in laboratories than the parent compounds. The bioavailability of UN-1 and UN-2 increased slightly and the drug score increased for UN-2 and UN-3 (Table 3).

Upon testing the ADMET properties, we found that all the designed inhibitors exhibited similar and, in some cases better, binding affinities towards SARS-CoV-2 SGp and M^pro^ active sites. Most of the interacted residues of SGp with the three inhibitors include Arg454, Arg457, Lys458 and Ser459 (Table S2†). Previous research showed that SARS-CoV-2 may interact with the host cell surface glycosaminoglycans (GAG) through SGp to invade the host cell and it contains three putative GAG-binding motifs where the 453–459 sequence consists of site 1.^42^ This endorses the theory that the designed compounds may interfere with the interaction of GAGs by binding with the GAG-binding motif. Several residues of SGp variants including the mutants interacted with the designed inhibitors. The mutant residues formed hydrogen bonds and carbon–hydrogen bonds with the inhibitors, indicating strong destabilization of the residues, thus preventing their binding with ACE2. In our docking analysis with M^pro^, most of the compounds interacted with Met49, His163, Cys145 and Met165 (Table S2†). All the inhibitors bind with Cys145 which is a residue of a catalytic dyad of M^pro^ located in a cleft between the two domains. UN-2 and UN-3 each formed a hydrogen bond with CYS145. All the other interacted residues with the designed inhibitors serve as substrate binding residues of M^pro^.

Finally, MD simulation results confirmed the stability of the three designed inhibitors with SGp, SGp variants and M^pro^. The RMSD plot suggests that all three inhibitors are stable and the RMSD values did not show any sudden surge throughout the simulation period (Fig. 7). RMSF analysis confirmed that the complexes showed less fluctuation in an acceptable range. The radius of gyration (*R*_g_) of all the protein–inhibitor complexes tend to be in same manner, suggesting that each complex had relatively similar behavior of compactness (Fig. 8). The SASA values depicted that, except for the M^pro^-UN-3 complex, the remaining complexes did not significantly increase in volume. A good number of hydrogen bonds was observed in all ten complexes throughout the simulation, explaining their conformational stability one more time (Fig. 9). Further, the binding free energies were calculated using the MM/PBSA method for all the complexes and the results show that five complexes showed favorable binding energy except for the M^pro^-UN-2 complex. The SGp(L452R)-UN-1 complex showed the lowest binding energy among the others (Fig. 10). From the per-residue interaction energy profile, it can be concluded that residues Arg454, Arg457, Lys458, Ser469 and Pro491 of SGp and Met49, Cys145, Met165 and Gln189 of M^pro^ play an essential role in protein–inhibitor stabilization and make significant contributions to the binding of the designed inhibitors. Notable contributions of the mutant residues of the SGp variants were also observed, confirming that the inhibitors are capable of stabilizing the variants and interfering in stable binding with ACE2 (Fig. 10). Thus, these designed inhibitors have the ability to bind with several variants of SARS-CoV-2 and inhibit their activity.

As the present study has been conducted through intensive computational analysis, there might be some limitations. Although the three designed analogues showed tremendous results in inhibiting the two proteins, they have not performed in animal model experiments. So, adequate experimental wet lab validations are needed to confirm their therapeutic efficacy.

## Conclusion

Our study proposed, designed and tested three novel inhibitors, UN-1, UN-2 and UN-3 against the SGp and M^pro^ of SARS-CoV-2 and its variants. The binding of these inhibitors to the target proteins may eventually create hindrance in entering host cells and in viral replication. Hence, after further *in vitro* and *in vivo* experiments, these designed inhibitors might be probable candidates as plausible therapeutics against SARS-CoV-2.

## Funding

There are no sources of funding for this study.

## Data availability

We have used Online SMILES Translator and Structure File Generator https://cactus.nci.nih.gov/translate/); (Discovery Studio 4.0 client (http://www.accelrys.com/products/discovery-studio/); Topography of Protein (CASTp) (http://www.sts.bioengr.uic.edu/castp/) server which provides an online resource for locating, depicting and measuring concave surface regions on 3D structures of proteins.^26^ Osiris property explorer (https://www.organic-chemistry.org/prog/peo/); The ADMET (absorption, distribution, metabolism, excretion and toxicity) properties of the designed analogs were determined by pkCSM server^34^ (http://biosig.unimelb.edu.au/pkcsm/).

## Author contributions

Conceptualization and methodology was performed by MNR, NF and MUH; formal analysis and data curation was performed by MNR, NF and MTHE; writing – original draft prepared by MNR, NF and MUH; writing – review and editing performed by MNR, NF, MUH, AKMM and MSI; supervised by MUH. All authors have read and agreed to submit the final version of the manuscript.

## Conflicts of interest

No potential conflict of interest relevant to this article was reported.

## Supplementary Material

RA-011-D1RA04107J-s001
