# Supplementary material for: Newly designed analogues from SARS-CoV inhibitors mimicking the druggable properties against SARS-CoV-2 and its novel variants
Source: RSC Adv. 2021 Sep 22;11(50):31460–76. doi: 10.1039/d1ra04107j (PMC9041434; doi:10.1039/d1ra04107j)
Supplement: RA-011-D1RA04107J-s001 [file RA-011-D1RA04107J-s001.pdf]

Electronic Supplementary Material (ESI) for RSC Advances.
This journal is © The Royal Society of Chemistry 2021

Supplementary Table S1: Molecular docking analysis of seventeen phytochemicals against WT SGp (6LZG) and M^{pro} (6LU7).

Phytochemicals (PubChem Compound CID)	Binding energy with SGp (kcal/mole)	Binding with active site residues of SGp	Binding energy with M^{pro} (kcal/mole)	Binding with active site residues of M^{pro}
Aloe-emodin (10207)	-6.1	Arg454, Phe456, Arg457, Lys458, Asp467, Glu471	-7.5	His41, Leu141, Asn142, Cys145, Glu166, Arg188,
Amentoflavone (5281600)	-7.9	Asp467, Asp471	-10.1	Thr26, His41, Met165, Glu166, Asp187
Apigenin (5280443)	-6.2	Arg457, Asp467, Glu471	-7.7	His163, His164, Leu141, Ser144, Glu166, Asp187
beta-Sitosterol (222284)	-5.5	No binding occurred with any residue of active site	-5.2	No binding occurred with any residue of active site
Betulonic acid (122844)	-6.4	No binding occurred with any residue of active site	-7.1	Tyr54
Curcumin (969516)	-5.5	Tyr473	-5.4	Thr24, Thr25, Cys44, Thr45
Hesperetin (72281)	-5.9	Glu471, Ile472, Gln474	-7.0	Met49, Leu141, Ser144, His163, Met165, Glu166
Hinokinin (442879)	-6.3	Arg457, Lys458, Asp467	-7.2	No binding occurred with

				any residue of active site
Indigo (10215)	-6.9	Arg454, Arg457, Lys458, Asp467, Glu471, Pro491	-7.0	No binding occurred with any residue of active site
Isotheaflavin 3'-Gallate (135704008)	-7.2	Arg454, Arg457, Lys458, Asp467, Ser469	-7.2	Thr26, His41, Ser46, Ser144, Leu141, Asn142, Cys145, His163, Glu166
Luteolin (5280445)	-7.0	Arg454, Lys458, Asp467, Ser469, Glu471, Arg457	-7.4	His41, Asn142, Cys145, Arg188, Thr190, Gln192
Myricetin (5281672)	-6.3	Arg454, Lys458, Arg457, Asp467, Glu471	-7.4	Leu141, Gly143, Ser144, Cys145, His163, Met165
Niclosamide (4477)	6.2	Arg454, Arg457, Lys458, Asp467, Ser469, Glu471	-7.0	Thr26, Leu141, Gly143, Ser144, Cys145, Glu166
Quercetin (5280343)	-6.3	Arg454, Arg457, Lys458, Asp467, Ser469, Glu471	-7.3	Leu141, Gly143, Ser144, Cys145, His163, Met165, Gln180, Arg188
Savinin (5281867)	-6.5	Glu471	-7.3	Leu141, Ser144, Glu166
Scutellarein (5281697)	-6.1	Arg457, Asp467, Glu471	-8.0	Tyr54, Phe140, Leu141, Ser144, His163, His164, Asp187
Theaflavin 3,3'-	-6.5	Arg454, Phe456,	-8.9	His41, Ser46,

Digallate (135403795)		Arg457, Lys458, Asp467, Ser469, Glu471		Leu141, Gly143, Ser144, Cys145, Met165, Glu166, Gln189, Thr190

Supplementary Table S2: Physicochemical and ligand binding properties of luteolin, myricetin, quercetin and the three designed inhibitors.

**Properties**	**Luteolin**	**UN-1**	**Myricetin**	**UN-2**	**Quercetin**	**UN-3**
Formula	$C_{15}H_{10}O_6$	$C_{16}H_{11}NO_4$	$C_{15}H_{10}O_8$	$C_{16}H_{11}NO_4$	$C_{15}H_{10}O_7$	$C_{15}H_{11}NO_4$
Molecular Weight (g/mol)	286.24	281.26	318.24	281.26	302.24	269.25
Consensus Log $P_{o/w}$*	1.73	2.10	0.79	2.36	1.23	2.05
Number of Rotatable Bonds	1	2	1	2	1	1
Number of hydrogen bond acceptors	6	4	8	4	7	4
Number of hydrogen bond donors	4	3	6	3	5	4
TPSA (Å²)	111.13	90.39	151.59	90.39	131.36	93.55
Docking energy with WT SGp (kcal/mole)	-7.0	-7.0	-6.3	-5.9	-6.3	-6.5
Interacting	Arg454,	Arg457,	Arg454,	Arg454,	Arg454,	Arg457,

residues of WT SGp	Arg457, Lys458, Asp467, Ser469, Glu471	Lys458, Ser459, Asp467, Glu471	Arg457, Lys458, Asp467, Glu471	Arg457, Lys458, Asp467, Glu471, Pro491	Arg457, Lys458, Asp467, Ser469, Glu471	Lys458, Ser459, Asp467, Glu471
Docking energy with M^{pro} (kcal/mole)	-7.4	-7.5	-7.4	-6.9	-7.3	-7.9
Interacting residues of M^{pro}	His41, Asn142, Cys145, Arg188, Thr190, Gln192	Met49, Tyr54, Cys145, Met165, Gln189	Leu141, Gly143, Ser144, Cys145, His163, Met165	Gly143, Ser144, Cys145, Met165, Arg188	Leu141, Gly143, Ser144, Cys145, His163, Met165, Arg188	Met49, Leu141, Ser144, Cys145, His164, Glu166

*Consensus Log Po/w is the average value of iLOGP, XLOGP3, WLOGP, MLOGP and SILICOS-IT

Supplementary Table S3: Ligand binding properties of the three designed inhibitors with SGp variants.

Mutation site	Designed inhibitor	Binding energy (Kcal/mol)	Interacted residues
K417N	UN-1	-5.8	Arg403, Glu406, Asn417, Tyr453, Tyr495
	UN-2	-5.6	Arg403, Asn417, Tyr421, Tyr453
	UN-3	-5.9	Arg403, Asn417, Tyr453, Tyr501
	UN-1	-6.3	Lys484, Tyr489, Phe490,

E484K			Leu492
	UN-2	-6.1	Leu452, Lys484, Tyr489, Leu492
	UN-3	-5.6	Phe456, Lys484, Tyr489, Phe490, Gln493
N501Y	UN-1	-6.4	Arg403, Tyr449, Tyr453, Ser494, Tyr501
	UN-2	-6.8	Arg403, Tyr453, Tyr501, Tyr505
	UN-3	-6.4	Tyr453, Ser494, Tyr495, Tyr501, Tyr505
L452R	UN-1	-6.7	Arg452, Tyr489, Phe490, Leu492, Gln493
	UN-2	-6.2	Arg452, Leu492, Gln493
	UN-3	-6.1	Arg452, Phe490, Leu492

Supplementary Table S4: Per residue contribution energy of SGp (WT and mutants) withUN-1, UN-2 and UN-3.

**WT SGp**				**SGp variants**			
				**K417N**	**E484K**	**N501Y**	**L452R**
**Residue number**	**Contributed energy of UN-1**	**Contributed energy of UN-2**	**Contributed energy of UN-3**	**Contributed energy of UN-3**	**Contributed energy of UN-1**	**Contributed energy of UN-2**	**Contributed energy of UN-1**
333	-6.8604	-6.8846	-0.0187	-	-	-	-6.0608
334	0.0136	0.06	-0.0007	-0.1033	-8.965	-7.8207	0.0218
335	0.0114	0.0178	0.0003	-0.0057	0.0578	0.0439	0.0444
336	-0.0327	-0.0101	0.0005	0.0014	0.0825	0.0243	0.0209

337	0.0787	0.087	-0.0011	-0.0098	0.0888	0.0364	0.049
338	-0.0228	0.131	-0.0019	-0.0097	0.1877	0.0351	0.0829
339	0.0197	0.135	-0.0003	-0.01	0.0037	0.0936	0.0006
340	9.2874	8.7097	0.0416	-0.0282	15.9315	10.6362	8.1149
341	0.1306	0.1643	-0.0051	-0.0185	0.2017	0.139	0.1124
342	0.0562	0.1567	-0.0017	-0.0252	0.2269	0.1832	0.1434
343	0.0575	-0.0412	-0.0022	-0.0139	0.1831	0.1925	0.1011
344	-0.0376	-0.0529	0.0011	0.0057	0.1524	0.0788	0.0297
345	0.117	0.0599	0.0035	0.0019	0.7542	0.2135	0.1324
346	-10.6383	-9.3467	-0.087	0.1443	-25.3479	-14.0938	-9.2079
347	0.1104	0.0389	0.0012	-0.0036	0.1857	0.2543	0.0482
348	0.2337	-0.0021	-0.0068	0.0036	-0.0014	0.1098	0.0421
349	-0.0134	0.1426	-0.0185	-0.0081	-0.1125	-0.3493	-0.1356
350	0.0728	0.1981	-0.0434	-0.0036	-0.2944	-0.3961	0.1632
351	-0.1319	0.404	-0.1445	-0.0103	-0.7698	-0.4337	0.0317
352	-0.1139	-0.0886	-0.0029	0.0015	0.0187	-0.0575	-0.1129
353	-0.1153	-0.3041	0.0391	0.0021	0.1127	-0.0827	-0.1069
354	-0.0823	-0.1369	0.0154	0.0199	-0.293	0.0155	-0.008
355	-12.7524	-13.0288	0.089	0.5834	-11.5796	-10.5577	-9.8959
356	-11.1892	-9.5242	-0.0442	0.2408	-15.0489	-10.6277	-8.6535
357	-9.5504	-9.8472	-0.008	0.2917	-10.0207	-8.8294	-8.0895
358	-0.0332	-0.0001	-0.0005	-0.0043	-0.0382	-0.0166	-0.0119
359	0.0044	0.0122	0.0002	0.0038	-0.0743	-0.0374	-0.0314
360	-0.0312	-0.0108	0.0007	0.0167	0.0558	0.0382	-0.0313
361	0.041	0.0312	-0.0005	0.0016	0.0085	-0.0023	0.0197
362	0.0161	0.0251	0.0002	-0.0033	0.0886	0.0719	0.0263
363	-0.0552	-0.0189	-0.0001	0.0061	-0.04	-0.03	-0.0346
364	7.4681	7.43	0.0135	0.2928	9.6542	8.0581	6.9305
365	0.066	0.1181	-0.0015	0.0177	0.2232	0.1542	0.1085
366	0.0417	0.047	-0.0004	0.0144	0.1146	0.1281	0.01

367	0.0683	0.0581	-0.0002	0.0057	0.1727	0.1398	-0.002
368	0.0998	0.1195	-0.0012	-0.0085	0.2151	0.1677	0.0242
369	-0.0399	0.0788	-0.0001	-0.0075	0.1872	0.1584	-0.0483
370	-0.0256	0.0882	-0.0002	-0.0287	0.0665	0.1025	0.0032
371	0.0597	0.0344	-0.001	-0.0462	-0.0358	0.088	0.0155
372	0.0842	0.1009	-0.0005	-0.0205	0.1278	0.0016	0.0318
373	0.099	-0.0244	-0.0007	-0.0218	0.2474	0.0804	0.0613
374	-0.0269	-0.0017	0.0004	0	0.0321	-0.01	-0.0152
375	-0.0458	0.04	0.001	0.0173	-0.1584	-0.1048	0.0012
376	0.0443	0.0251	-0.0017	-0.0499	0.0126	0.0249	0.0057
377	-0.0013	0.018	-0.0005	-0.0963	-0.0469	-0.0174	-0.0319
378	-10.0307	-11.3876	0.0299	0.6305	-11.3869	-11.6579	-10.5657
379	0.005	-0.012	0.0001	-1.1181	-0.0317	-0.0426	-0.0165
380	-0.0204	-0.0502	-0.0007	-11.2869	-0.0304	-0.0374	-0.0285
381	-0.0765	-0.0786	0.0015	-2.0383	-0.0654	-0.0754	-0.0907
382	-0.0078	-0.0197	-0.0002	-2.5899	0.0371	0.0039	0.0146
383	-0.021	-0.0317	-0.0009	-1.9862	0.0369	0.0319	-0.0274
384	-0.0172	-0.0286	0.0007	-3.1035	0.002	-0.0066	-0.016
385	-0.0181	0.0022	-0.0009	-0.1951	-0.0093	-0.0362	-0.0698
386	-7.1038	-6.453	-0.0098	-1.1434	-7.5709	-7.9323	-6.5417
387	-0.009	0.046	0.0003	-0.1607	-0.0229	-0.0233	-0.0206
388	-0.0606	0.032	-0.0008	0.0456	0.0383	0.0046	0.0445
389	6.7599	8.4297	0.0029	0.7767	7.4506	6.7807	6.283
390	0.0355	-0.0317	-0.0003	-0.0422	0.0593	0.0006	0.0213
391	0.0055	0.0097	-0.0004	-0.0156	-0.0095	-0.0136	0.0165
392	0.0602	-0.073	0.0004	-0.0471	0.0136	-0.042	-0.0408
393	-0.0026	-0.0843	0.0007	0.0026	0.0403	-0.0258	-0.011
394	-0.0122	-0.0933	0.0007	0.0144	0.078	-0.0146	-0.0575
395	0.017	-0.0045	0.0009	-0.0084	0.0331	0.0038	0.0028
396	0.0669	0.03	-0.003	-0.0043	0.0259	0.0264	0.0359

397	0.0639	0.0103	0.0005	-0.0084	0.1336	0.0703	0.0167
398	12.6286	12.6554	-0.1056	-0.4125	12.3064	11.4478	10.5932
399	0.1143	0.1304	0.0002	-0.0051	0.2421	-0.0058	0.0348
400	-0.047	-0.0341	-0.0025	0.0018	-0.0434	-0.0566	-0.0336
401	0.0567	0.062	-0.0083	0.0004	-0.0332	0.0423	0.127
402	-0.0929	-0.0941	0.003	-0.0059	0.015	0.0144	-0.1166
403	-13.9485	-13.0523	0.1585	0.0583	-21.7503	-24.722	-19.594
404	0.0116	0.1099	-0.0066	-0.0188	-0.3219	-0.3542	-0.1017
405	13.2306	13.6088	-0.1969	-0.0775	15.9197	20.7535	18.0516
406	14.2265	14.3182	-0.2661	-0.1823	17.3365	20.2391	18.0851
407	0.0133	0.1447	-0.006	-0.0843	-0.2999	-0.3535	-0.1725
408	-11.1314	-11.8897	0.0871	-0.3263	-14.2472	-15.63	-13.7107
409	0.2594	0.7251	-0.0729	-0.1241	-0.167	-0.5663	-0.1711
410	-0.0978	0.1787	-0.0075	-0.1504	-0.1751	-0.1898	-0.1685
411	0.0238	0.1545	-0.0067	-0.5056	0.0382	0.003	0.0156
412	-0.1478	-0.1948	0.0065	-4.6012	-0.0698	-0.0405	-0.0172
413	-0.0252	0.0056	0.0044	-1.7324	0.0538	-0.0395	0.0858
414	0.0207	0.0975	-0.0118	-0.3612	0.0167	0.0513	0.0607
415	0.3675	0.2205	0.0023	-0.0983	0.0192	0.1693	0.0711
416	0.041	-0.0336	-0.0259	-0.0228	0.1214	-0.0108	-0.1168
417	-18.1515	-21.7094	0.5062	-0.0245	0.219	-0.349	-23.9931
418	0.1243	0.1029	-0.213	-0.0193	-0.248	-0.0259	-0.1205
419	0.0678	-0.085	-0.0754	-0.0258	-0.0968	-0.1868	-0.2325
420	18.6308	24.5654	-0.8518	-0.8076	12.805	12.0637	17.9335
421	-0.2925	-0.0177	-1.4627	-0.0108	0.0607	-0.137	-0.0393
422	-0.1655	-0.1846	-0.2268	-0.0194	0.2338	0.004	-0.4598
423	-0.1388	-0.0413	0.0192	-0.0479	-0.2223	-0.0857	-0.03
424	-17.103	-21.1614	0.5077	1.0729	-11.9297	-11.1722	-13.5082
425	0.061	0.1719	-0.0068	-0.4273	-0.074	-0.0495	0.0157
426	-0.1529	-0.1962	-0.0021	-0.546	0.0052	0.0412	-0.0214

427	13.6456	15.9648	-0.1221	-1.2183	10.0229	9.8923	10.6566
428	11.3141	12.2847	-0.0483	-1.8472	9.0096	8.9031	8.9059
429	-0.0367	-0.1023	-0.0012	-1.9259	-0.0414	-0.0443	-0.0291
430	-0.0101	0.0207	0.0003	-0.6983	-0.0142	-0.0325	-0.0296
431	0.0038	0.0319	0	-0.2587	-0.0079	0.0102	0.0198
432	0.044	0.0336	-0.002	-0.1702	0.0518	0.0445	0.0286
433	-0.0188	-0.01	0.0004	-0.1296	-0.0001	0.0514	0.0035
434	0.0296	0.0048	-0.0001	-0.0352	0.0879	0.0768	0.0122
435	-0.0544	-0.0403	0.0012	0.0013	-0.0484	-0.0416	-0.0374
436	0.1328	0.0845	-0.0033	-0.0035	-0.0442	0.0613	0.1262
437	0.0019	-0.0139	0.0024	-0.007	0.1417	-0.0962	-0.0168
438	-0.0261	-0.1049	-0.0015	-0.0036	0.0092	0.0535	-0.0303
439	0.0502	-0.0208	0.0026	-0.012	-0.0641	0.2464	0.0802
440	0.035	-0.0053	0.0002	-0.0099	0.3546	-0.0094	0.0758
441	0.048	-0.0363	0.0016	-0.0003	0.2369	0.0731	0.1051
442	10.4189	9.8658	0.0357	-0.1196	21.8324	19.9994	11.3464
443	-0.0185	-0.0443	0.0027	0.0013	0.2073	-0.1832	-0.0766
444	-10.9447	-8.6262	-0.0507	0.0801	-19.2625	-21.2943	-10.6105
445	0.1876	0.0757	0.0008	-0.0006	-0.5913	-0.8753	0.066
446	-0.0565	0.0556	-0.0045	0	-1.141	-1.0992	0.1879
447	-0.0801	0.0556	0.0018	0.0006	-1.7471	-1.3011	0.2585
448	0.1083	0.0809	-0.0046	-0.0048	-2.6966	-1.5863	0.1562
449	0.0472	-0.0098	-0.0061	-0.0004	-13.4411	-10.8878	-0.1276
450	0.0035	0.1015	-0.0077	0.0057	-1.7885	-0.7166	0.104
451	-0.0409	0.1194	0.0002	-0.0043	-0.4501	-0.1189	0.2879
452	-0.0031	0.0527	-0.0917	-0.0001	-3.3158	-0.7742	-16.3539
453	0.0674	0.0151	-0.0492	0.0011	-0.2109	-0.0868	-0.0346
454	-23.3	-19.4808	0.6404	0.2746	-15.5408	-15.4797	-16.8163
455	-0.2127	0.1351	-0.4256	-0.0185	-0.2726	-0.3484	0.064
456	0.4762	-0.5195	-7.16	-0.0009	-0.1204	0.1154	-1.9545

457	-26.9792	-27.6262	-3.6108	0.4646	-13.2835	-13.2528	-15.9549
458	-8.5855	-26.9006	-0.8259	0.352	-12.0827	-12.4639	-20.0934
459	-0.0828	-1.7522	-0.8901	-0.0004	0.0047	0.0837	-0.3431
460	0.7173	0.5238	-0.1793	-0.0039	0.077	0.0451	-0.3929
461	-0.5099	-0.0064	-0.1908	-0.0241	-0.1036	-0.1197	-0.0854
462	-21.3398	-22.9027	-0.0045	0.7898	-10.998	-10.3355	-10.8167
463	0.06	-0.0194	0.0178	-0.0285	-0.0625	-0.0606	-0.1037
464	-0.0682	-0.0024	0.0383	-0.0316	-0.0571	0.0595	-0.0692
465	18.113	21.9958	-0.2528	-0.6215	12.8743	11.165	10.8308
466	-13.8911	-14.1711	-0.0094	0.3873	-13.7969	-13.512	-11.3211
467	31.8574	23.9594	2.4577	-0.442	14.1255	13.8579	14.49
468	-0.9263	-0.6003	-1.0708	-0.0005	-0.0481	0.2716	0.1319
469	-2.4905	-0.4765	-3.1009	0.0086	-0.0477	0.1397	0.3268
470	-2.2447	-0.0967	-1.3825	0.0043	-0.1067	-0.0093	0.2791
471	28.3747	17.4332	5.5357	-0.4126	13.1251	13.8586	14.7921
472	-4.0215	0.4988	-3.6697	-0.0064	-0.141	-0.1983	-0.4632
473	-9.3327	-1.4926	-9.4263	0.0003	0.2392	0.0558	-0.0934
474	-6.3474	-4.8853	-0.5352	-0.0015	0.0518	0.1383	-0.1089
475	-0.5268	-2.9848	-0.3111	0.002	-0.0565	-0.1026	-1.3041
476	0.3552	-5.1018	-0.0532	-0.0048	0.0167	-0.19	-1.2746
477	0.4066	-5.6983	-0.0296	0.0055	0.0377	-0.3464	-1.4628
478	-0.3746	-0.5688	0.0104	-0.0067	0.1455	0.0996	-0.4046
479	-5.0448	-1.4037	-0.1421	0.0032	0.0711	0.0194	-0.0996
480	-2.6996	-0.4452	-0.3247	0.0065	0.072	0.0306	-0.3317
481	-0.8506	-0.0567	-0.0088	0.0118	0.1512	-0.0312	-0.1342
482	-0.4022	-0.1327	0.0165	0.004	-0.3394	0.1696	0.1574
483	-0.1231	0.13	-0.1165	-0.0026	-1.826	0.2558	0.0436
484	21.1061	16.2525	0.2554	0.2219	-19.0742	-12.0658	-0.6802
485	-0.2647	-0.3041	0.009	-0.0009	-0.0963	0.0669	-2.3431
486	-0.2248	-0.1865	-0.0237	-0.0054	-0.1731	-0.0033	-11.2788

487	0.2129	0.6128	-0.0854	0.0048	-0.1758	-0.1747	-1.8446
488	-0.6218	-0.2691	-0.2177	0.0055	0.2096	0.0883	-1.6343
489	0.0459	-0.0072	-0.4643	-0.0037	-0.1195	-0.9501	-7.6717
490	-0.5477	0.1277	-0.3642	0.0038	-5.8605	-1.5258	-0.1997
491	-3.1726	-0.2073	-5.9329	-0.0004	-0.7786	-0.0498	0.0482
492	-0.3331	-0.1957	-1.0922	-0.0006	-2.3958	-0.0945	0.0111
493	-0.2536	-0.015	-0.2021	-0.0081	-2.1084	-2.0788	-0.4694
494	-0.012	0.0637	-0.0329	0.0058	-2.0056	-0.2361	0.4383
495	-0.209	-0.1641	-0.0296	0.0023	-0.0734	-0.8955	0.2611
496	-0.0829	-0.1083	-0.0057	0.0044	-0.5872	-0.614	-0.2027
497	-0.0403	-0.0833	-0.0011	-0.0014	-0.1581	-0.1352	-0.1018
498	-0.0115	0.0444	-0.0024	0.0056	-0.1145	-0.4726	-0.1893
499	-0.0455	-0.0474	-0.0013	-0.0006	-0.2767	0.177	-0.0708
500	-0.0031	0.0128	-0.0008	0.0009	-0.0871	-0.0297	-0.0195
501	-0.0008	0.0098	-0.0057	0.008	-0.0389	-0.0332	0.0832
502	0.0185	0.0439	-0.0016	-0.004	-0.1201	-0.1604	0.1223
503	0.082	0.0159	-0.0039	0.0009	0.179	-0.1705	0.1622
504	0.1632	0.1367	-0.0101	-0.0071	-0.0237	-0.1157	0.2667
505	0.0912	-0.0144	-0.0065	-0.0064	0.0632	-1.2206	0.0514
506	0.0678	0.0703	-0.0067	-0.0014	-0.4217	-0.1141	0.1303
507	-0.1322	-0.1333	0.0029	0.0032	-0.0536	-0.1875	-0.1607
508	0.0488	0.0534	-0.0043	-0.01	0.0679	-0.0675	0.0763
509	-9.2431	-9.4762	-0.0182	0.0622	-18.9057	-15.6284	-10.1637
510	0.0203	-0.009	-0.0004	-0.0147	0.0132	-0.03	-0.0314
511	-0.0245	-0.0046	-0.0012	-0.0071	-0.0555	-0.0455	-0.0179
512	-0.0114	0.0165	0	-0.0356	-0.047	-0.0748	-0.0489
513	-0.0342	-0.013	-0.0008	-0.0444	-0.0857	-0.0746	-0.0246
514	-0.0839	-0.0663	0.0017	-0.0232	-0.1149	-0.095	-0.0526
515	0.016	0.0806	-0.0004	-0.1155	-0.0139	-0.0317	-0.0075
516	9.4952	12.4141	-0.0105	-0.6109	9.2145	8.8689	8.5364

517	-0.0666	0.0538	0	-0.0024	-0.081	-0.0086	0.0016
518	-0.0188	-0.0213	-0.0005	0.0062	0.0188	-0.0219	-0.0289
519	0.0254	-0.1581	0.0005	0.0011	-0.0524	-0.0131	-0.1034
520	0.0272	0.0217	-0.0005	0.0064	0.0185	0.0063	0.0145
521	-0.039	0.0225	0.0001	-0.0116	0.0303	0.0537	-0.0241
522	-0.0044	-0.0724	0.0001	-0.0001	0.0397	-0.0169	0.0383
523	0.0153	-0.1144	0.0002	0.0128	0.0282	-0.0557	-0.009
524	0.0128	0.0259	-0.0007	0.0077	-0.0131	-0.001	0.0053
525	-0.0032	-0.006	0	-0.005	0.0035	0.0116	-0.013
526	-0.022	-0.0687	0.0002	0.4016	8.2308	7.1018	0.0043
527	6.8561	7.2825	0.0104	-	-	-	6.0883

Supplementary Table S5: Per residue contribution energy of M^{pro} with UN-1, UN-2 and UN-3.

Residue number	Contributed energy of UN-1	Contributed energy of UN-2	Contributed energy of UN-3
1	-9.564	-7.8702	0.012
2	0.0629	0.0022	0.0008
3	0.1031	0.0893	0.0006
4	-11.0755	-10.3296	0.0166
5	-13.3179	-12.1276	0.1876
6	0.031	0.0469	-0.0048
7	-0.1058	-0.0437	0.0082
8	0.09	-0.0316	-0.0036
9	-0.0437	0.0514	-0.0038
10	-0.0621	0.011	-0.0057
11	0.1128	0.0447	-0.0073
12	-10.4096	-8.9497	-0.01
13	0.1813	0.0686	-0.0105
14	14.0701	9.914	-0.1342

15	0.0897	-0.0277	-0.0019
16	0.1022	0.0346	-0.0036
17	-0.0069	-0.0386	-0.0045
18	0.1665	0.1046	-0.0144
19	-0.2785	-0.0891	0.0119
20	0.2873	0.0821	-0.0094
21	-0.2154	-0.0286	0.0013
22	0.032	-0.1763	0.0114
23	0.1175	-0.1915	0.0253
24	0.3282	-0.3481	0.0296
25	-0.5126	0.1137	-0.0056
26	0.3866	0.1199	-0.0188
27	-0.4764	-0.5306	-0.0068
28	-0.97	-0.4095	-0.041
29	-0.0375	-0.054	0.0042
30	-0.1674	-0.082	0.0105
31	-0.0012	0.0143	-0.0025
32	-0.08	-0.0311	0.0057
33	10.9034	9.2424	-0.0298
34	11.0693	9.5109	-0.0113
35	0.0183	0.0143	-0.0048
36	0.0269	0.0598	-0.0105
37	0.1682	0.0923	-0.0142
38	-0.2364	-0.0365	-0.0203
39	-0.157	-0.0823	0.0059
40	-11.2756	-15.2817	1.8966
41	-6.8685	-1.0282	-0.2076
42	0.0798	-0.0619	0.0207
43	-0.2295	-0.1344	0.0106
44	-0.5536	-0.2342	-0.1595

45	-0.5959	0.0145	-0.0188
46	-1.2242	-1.2097	-0.2586
47	23.6942	21.9786	3.8424
48	18.5272	19.2127	-4.3757
49	-9.2721	-5.0496	-11.4018
50	-1.2017	-5.0514	-5.8372
51	-0.4828	-0.5079	-1.3894
52	-2.1833	-0.1311	-3.9727
53	-0.2197	-0.2739	-0.1297
54	-0.8888	-0.0607	0.0738
55	13.7017	12.962	-0.3359
56	13.8278	13.5425	-0.2038
57	-0.1142	-0.2153	0.0435
58	-0.0129	-0.1531	0.0308
59	-0.104	-0.1745	0.0248
60	-14.6808	-13.334	0.1102
61	-18.6463	-14.1395	0.0979
62	0.0895	0.0008	-0.0082
63	0.3089	0.1298	-0.018
64	0.0783	-0.0619	0.0061
65	0.494	0.1605	-0.0137
66	-0.13	-0.093	0.0068
67	0.1268	0.0391	-0.0066
68	-0.0837	-0.0549	0.0047
69	-0.1579	-0.0213	-0.0043
70	-0.1011	-0.0554	0.0022
71	0.0802	0.0324	0.0026
72	0.2702	0.072	-0.0006
73	0.0322	0.0527	-0.0051
74	-0.1389	-0.0733	0.0014

75	0.1033	0.0265	-0.0054
76	-12.6976	-9.0858	-0.0211
77	-0.1434	-0.0577	0.0071
78	-0.1014	-0.0661	0.007
79	0.0163	-0.0021	-0.007
80	-0.0198	-0.0563	0.0019
81	0.0684	0.0612	-0.0208
82	-0.0721	-0.0329	0.0091
83	0.1584	-0.0115	-0.0307
84	-0.2122	0.2289	-0.1872
85	0.2689	0.2323	-0.096
86	-0.1238	-0.008	0.0037
87	-0.1161	-0.0575	0.0077
88	-12.4029	-10.9322	0.2299
89	-0.0696	-0.0645	0.0064
90	-11.0071	-9.5085	-0.0245
91	-0.0554	-0.0459	0.003
92	10.2916	8.8907	0.0376
93	0.0792	0.0418	-0.006
94	0.026	0.0054	0.0006
95	-0.2461	-0.1978	0.0185
96	-0.0913	-0.0624	0.0015
97	-11.8306	-8.6595	-0.0153
98	-0.0785	-0.0796	0.0071
99	0.0147	0.014	-0.0005
100	-9.6362	-8.3844	-0.0351
101	-0.0175	0.0112	0.0057
102	-10.6395	-9.6906	-0.0004
103	-0.0589	-0.0034	0.0083
104	0.0821	0.0806	-0.0137

105	-12.5094	-12.8379	0.4437
106	-0.1111	-0.0022	-0.0015
107	0.0517	0.0305	-0.0199
108	-0.1279	-0.1392	0.0167
109	-0.0569	-0.096	0.0116
110	0.2749	0.331	-0.0326
111	-0.0302	-0.0394	0.0004
112	0.0625	-0.0126	-0.0009
113	0.0167	0.02	0.0033
114	0.0463	-0.0246	0.0061
115	0.2982	0.1188	-0.0277
116	-0.0392	-0.0863	0.0177
117	0.5064	0.1308	-0.0063
118	-0.3794	-0.0656	-0.004
119	-1.3452	-0.4443	0.0334
120	-0.2706	-0.2284	0.0028
121	0.075	0.0451	0.0001
122	-0.3174	-0.0885	0.0136
123	0.0792	-0.0466	-0.0057
124	0.3709	0.1203	-0.0364
125	-0.0363	-0.0222	0.0071
126	-0.0111	0.047	-0.0192
127	0.253	0.1251	-0.0252
128	-0.0669	-0.07	0.003
129	0.1177	0.1249	-0.0287
130	-0.1083	-0.0394	0.0101
131	-15.2927	-16.049	0.4202
132	-0.0057	0.043	-0.0175
133	0.1638	0.576	-0.0616
134	0.1792	0.051	-0.1221

135	-0.1842	0.0026	-0.024
136	0.012	0.1463	-0.0724
137	-13.0733	-15.9727	1.1251
138	-0.0196	-0.0904	-0.0311
139	-0.0524	-0.1187	-0.0173
140	-0.0454	0.1543	-0.1168
141	0.2278	-0.0745	-0.0282
142	-0.4244	-0.705	-0.0269
143	-0.7825	-0.9252	-0.0464
144	0.0506	-1.0616	-0.0211
145	-0.4684	-1.2328	-0.2053
146	-1.0277	-0.2735	0.0707
147	-0.6112	-0.1862	0.01
148	-0.0843	-0.0445	-0.0017
149	-0.1226	-0.0503	0.0108
150	-0.0394	-0.002	-0.0036
151	-0.0924	-0.0513	0.0074
152	-0.0504	-0.0421	0.0039
153	10.0951	9.1811	-0.0103
154	0.0297	-0.0276	0.0008
155	9.6911	8.4148	0.0171
156	0.0395	0.0331	-0.0032
157	0.0384	0.0348	-0.0029
158	0.082	0.0588	-0.0113
159	-0.0118	0.0444	-0.0021
160	0.0135	0.0687	-0.0062
161	-0.0491	0.0072	0.0173
162	-0.1378	0.0736	-0.0449
163	-0.4856	-0.6831	-0.0036
164	-0.7945	-0.0547	-0.2845

165	-10.23	-3.8338	-5.0873
166	28.2601	20.6628	-7.0413
167	-1.0422	-4.2442	-6.6024
168	-0.5743	-0.9196	-7.9452
169	-0.1695	-0.3793	-0.6601
170	-0.1286	-0.4501	-0.1489
171	0.1019	0.1711	-0.3915
172	-1.171	-0.203	-0.1477
173	-0.1583	-0.3873	-0.3773
174	-0.2087	0.0072	-0.0636
175	0.0884	-0.1962	-0.0718
176	13.7534	13.6332	-0.5113
177	-0.016	-0.0632	-0.0057
178	12.1567	11.5613	-0.0932
179	-0.1357	-0.0948	0.014
180	0.0471	-0.0095	0.0156
181	-2.4087	-0.0984	-0.0414
182	-0.1202	0.0464	-0.0377
183	0.1441	0.1368	-0.1559
184	0.1284	-0.0321	-0.0252
185	-0.7508	-0.3817	-1.1175
186	-0.6552	-0.1941	-0.1097
187	12.5049	16.4422	-0.9314
188	-24.1413	-21.3162	0.7304
189	-8.1295	-6.3937	-4.4563
190	-3.1927	-2.9884	-3.9538
191	-2.793	-1.1945	-1.8221
192	-0.8308	-0.0818	-0.3576
193	-0.1548	-0.0358	-0.0539
194	-0.0799	0.1569	0.0178

195	0.0361	-0.2622	0.0145
196	0.0136	-0.5138	-0.0227
197	13.6278	18.3187	-0.3068
198	0.1364	0.1729	-0.019
199	-0.0754	0.0598	0.0117
200	-0.0714	-0.143	0.007
201	-0.0065	-0.2511	-0.0024
202	-0.1077	-0.2137	0.0041
203	-0.2366	-0.2087	0.0156
204	-0.081	-0.187	0.0053
205	-0.1025	-0.1778	0.0041
206	-0.1045	-0.1748	0.0052
207	-0.1419	-0.1529	0.003
208	-0.0909	-0.1485	0.0035
209	-0.0611	-0.0773	0.0028
210	-0.0962	-0.1363	0.0038
211	-0.0873	-0.1231	0.0029
212	-0.0947	-0.1152	0.002
213	-0.1013	-0.1143	0.0019
214	0.0021	-0.0879	-0.0004
215	-0.045	-0.0626	0.0006
216	7.8463	7.8685	-0.0396
217	-7.3771	-7.2236	0.0233
218	-0.1443	-0.0848	0.0021
219	-0.0344	0.0381	-0.0003
220	-0.0563	-0.0443	0.0008
221	-0.0588	-0.0297	0.0009
222	-6.8688	-7.3858	0.0199
223	0.013	0.0198	-0.0001
224	0.0014	0.0226	0.0005

225	0.0206	0.0351	0.0005
226	0.0505	0.0664	-0.0014
227	0.0786	0.1437	-0.003
228	0.1361	0.2655	-0.0029
229	7.4089	10.0296	-0.0338
230	0.0584	0.1254	-0.0034
231	0.1327	0.1861	-0.0084
232	0.0964	0.2484	-0.0039
233	0.0748	0.1751	-0.0045
234	0.0958	0.1463	-0.0074
235	0.1293	0.2755	-0.0084
236	-7.9301	-11.7968	0.0578
237	0.0629	-0.0251	-0.0048
238	0.0882	-0.096	-0.0085
239	-0.0196	0.1093	0.0052
240	11.2593	16.2257	-0.1125
241	0.0939	0.0858	0.0002
242	-0.0309	0.0158	0.0009
243	-0.0913	-0.1589	-0.0013
244	-0.0407	-0.1501	-0.0013
245	10.0133	11.5987	-0.0009
246	-0.2256	-0.2803	0.0014
247	-0.0437	-0.1405	-0.0008
248	8.7137	9.243	-0.0005
249	-0.0379	-0.1317	0.0001
250	-0.0404	-0.1196	-0.0003
251	-0.0849	-0.1104	-0.0001
252	-0.0706	-0.0774	0.0006
253	-0.0878	-0.112	0.0007
254	-0.0663	-0.0752	0.0008

255	-0.0881	-0.092	0.0003
256	-0.0451	-0.0498	0.0003
257	-0.1001	-0.0927	0.0004
258	-0.0657	-0.0425	0.0003
259	0.0388	0.0479	-0.0007
260	0.045	0.0565	-0.0016
261	0.0459	0.0699	-0.0027
262	0.0302	0.0736	-0.0021
263	7.0403	8.0149	-0.0246
264	0.0289	0.0503	-0.0024
265	0.0479	0.092	-0.0029
266	0.0193	0.0646	-0.0018
267	-0.0133	-0.0136	-0.0011
268	0.0295	0.0597	-0.0027
269	-7.3431	-9.4331	0.0406
270	7.1845	8.7751	-0.0451
271	0.0167	0.0289	-0.0015
272	0.0325	0.0843	-0.0029
273	0.0262	0.0864	-0.0019
274	0.0221	-0.0005	-0.0006
275	0.0082	-0.0862	0.0011
276	-0.0116	-0.0552	-0.0011
277	0.0178	-0.1321	0.0029
278	0.0001	-0.0398	0.0001
279	-7.4388	-7.7728	0.0417
280	-0.0758	-0.0559	0.0011
281	-0.118	-0.1517	0.0037
282	0.0768	0.045	-0.0013
283	0.0046	0.0114	-0.0008
284	-0.1562	-0.1461	0.0033

285	-0.1038	-0.0409	0.0009
286	0.0197	-0.048	-0.0015
287	0.1329	0.1582	-0.0068
288	11.6913	11.6593	-0.1389
289	12.0728	13.246	-0.2958
290	12.9122	11.2536	-0.3111
291	-0.0078	0.0271	0.0005
292	-0.1043	-0.1226	-0.0002
293	-0.0614	-0.0936	0.0012
294	-0.0985	-0.1546	0.0021
295	11.2621	10.2367	-0.0515
296	-0.0755	-0.1104	0.0035
297	-0.1086	-0.1227	0.002
298	-10.5228	-9.168	0.0035
299	-0.1245	-0.1728	0.0027
300	-0.1031	0.0169	0.0013
301	-0.0513	-0.0018	-0.0009
302	-0.0654	-0.0592	0.0002
303	0.0483	0.0232	-0.0003
304	0.0423	-0.0291	-0.0001
305	0.0595	-0.0114	-0.0002
306	9.887	6.6816	-0.0152
